# *In vitro* antiplasmodial activities of extract and fractions from *Lepidobotrys staudtii* against sensitive and resistant blood-stage parasites of *Plasmodium falciparum*

**DOI:** 10.1016/j.ijpddr.2025.100610

**Published:** 2025-08-25

**Authors:** Jeannette Nina Magoudjou Pekam, Noella Molisa Efange, Lakshminarayana Mishro, Rodrigue Keumoe, Bruno Lenta Ndjakou, Lawrence Ayong, Frédéric Nico Njayou, Paul Fewou Moundipa, Vinoth Rajendran

**Affiliations:** a Laboratory of Pharmacology and Toxicology, Department of Biochemistry, Faculty of Sciences, University of Yaoundé I, Yaoundé, Cameroon; b Malaria Research Unit, Centre Pasteur du Cameroun, P.O. Box 1274, Yaoundé, Cameroon; c Department of Microbiology, School of Life Sciences, Pondicherry University, Puducherry, 605014, India; d Department of Biochemistry and Molecular Biology, University of Buea, Cameroon; e Department of Chemistry, National Ayurveda Research Institute for Panchakarma, Cheruthuruthy, Kerala, India; f Antimicrobial and Biocontrol Agents Unit (AmBcAU), Laboratory for Phytobiochemistry and Medicinal Plants Studies, Department of Biochemistry, Faculty of Science, University of Yaoundé I, Yaoundé, Cameroon; g Higher Teachers Training College, University of Yaoundé I, Cameroon

**Keywords:** Malaria, *Lepidobotrys staudtii*, *Plasmodium falciparum*, Blood-stage, Parasite killing, Drug-combination

## Abstract

Antimalarial resistance is a primary challenge in the treatment of malaria. The ongoing search for novel drug sources remains a critical strategy for addressing this issue. This study evaluated the blood stage antiplasmodial and cytotoxic activities of the crude extract and fractions obtained from *Lepidobotrys staudtii*. The crude extract and all fractions exhibited promising antiplasmodial activity (IC_50_ < 10 μg/mL) against all the tested *Plasmodium falciparum* strains (*Pf*3D7 drug-sensitive and *Pf*INDO chloroquine-resistant). Notably, the hexane and ethyl acetate fractions exhibited the highest potency, with IC_50_ values of 3.73 and 3.4 μg/mL (*Pf*3D7), respectively. No cytotoxic effects were observed at concentrations of up to 500 μg/mL. The ethyl acetate fraction displayed rapid action (12 h of exposure) against the *Pf*3D7 and *Pf*INDO strains. The ring stage parasites were particularly susceptible to the fractions, with IC_50_ values ranging from 2.17 to 4.87 μg/mL (*Pf*3D7) and 2.27–6.27 μg/mL (*Pf*INDO). Additionally, combining the fraction with standard antimalarials at fixed sub-inhibitory concentrations significantly reduced IC_50_ values. Only the hexane and crude extracts stimulated reactive oxygen species (ROS) production, whereas the other fractions neutralized the ROS. The most potent ethyl acetate fraction arrested parasite developmental progression and merozoite egress. Phytochemical analyses revealed the presence of phenols, flavonoids, tannins, alkaloids, saponins, carbohydrates, glycosides, and proteins. Reverse Phase High Performance Liquid Chromatography (RP-HPLC) analysis revealed that the fractions comprised a diverse array of compounds, resulting in varying levels of parasite-killing. This study emphasizes the blood-stage antiplasmodial properties of the stem bark extract and fractions of *L. staudtii*, underscoring their potential as a promising source of antimalarial agents.

## Highlights

• Stem bark extract and its fractions from *Lepidobotrys staudtii* impede the development of *Plasmodium falciparum* parasites in their blood stages.
• The fractions or extracts demonstrated equal effectiveness in both sensitive and resistant strains.
• Parasites at the ring stage showed a high level of sensitivity to various fractions or extracts.
• Chloroquine had a synergistic effect with the fractions against the CQ-resistant strains.
• Fractions trapped reactive oxygen species produced during parasite invasion.

## Graphical abstract

**Figure:**
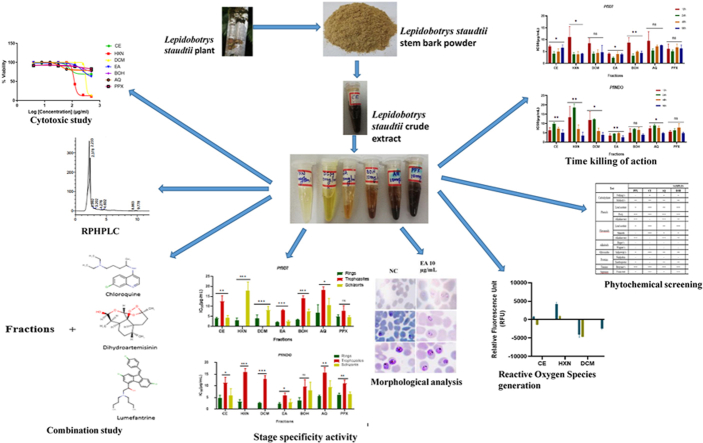

## 1 Introduction

There is significant interest in developing novel active compounds to combat malaria (Mishra et al., 2017; Ward et al., 2022), a parasitic disease caused by the protozoan *Plasmodium falciparum* (Kamdoum et al., 2022). Malaria is a major public health problem which is reported to be responsible of 249 million of hospital admission for about 608000 deaths, with 85 %, of cases found in the WHO African region. The emergence of drug resistance and lack of an effective vaccine complicate efforts to eradicate the disease (World Health Organization WHO, 2023).

The parasite's life cycle comprises various stages, among which the asexual phase, known as the blood stage, occurs in the human host (Kamdoum et al., 2022). Upon entering the erythrocytes, the parasites progress from the ring stage to the trophozoite stage, characterized by high metabolic activity, and ultimately to the schizont stage, which facilitates parasite replication owing to its multinucleated structure (Kavishe et al., 2017). During the erythrocytic cycle, the growth and development of the parasite are facilitated by the degradation of hemoglobin, which supplies the amino acid blocks necessary for DNA synthesis and creates space for parasite expansion within the erythrocyte (Rosenthal et al., 2002; Singh et al., 2020). The toxic free heme resulting from this process is transformed into inert hemozoin via polymerization reactions. However, many of these toxic heme molecules can escape this detoxification process and interact with membrane phospholipids. The Fe^2+^ ions contained in these molecules can catalyze Fenton and Haber-Weiss reactions, generating free radicals (Francis et al., 1997; Vasquez et al., 2021; Percário et al., 2012).

The erythrocytic phase is primarily responsible for the onset of symptoms and serves as the principal target for antimalarial drugs (Ariefta et al., 2023). Although the production of reactive oxygen species enhances the mechanism of action of certain antimalarial compounds, it can also have a negative impact on host function. Furthermore, the host antioxidant defense system cannot always restore oxidative balance following stress (Vasquez et al., 2021; Wagner et al., 2022). The use of antimalarial therapies capable of restoring the redox balance would help in antimalarial treatment.

Historically, chloroquine, a quinoline derivative, has served as a standard pharmacological intervention for malaria. Nevertheless, its perceived toxicity and emergence of resistance have rendered it ineffective, necessitating exploration of novel compounds (Pandey et al., 2023). Following its discovery, artemisinin has quickly become the preferred treatment for malaria because of its rapid action and efficacy. However, its short half-life and development of resistance in *P. falciparum* strains have diminished its effectiveness over time. The discovery of new molecules is essential for developing effective drugs against multidrug-resistant *Plasmodium* species. Plant-derived phytomolecules play a significant role in this search, as they offer a diverse range of bioactive compounds. These compounds can lead to the identification of novel therapeutic agents that target various biological pathways, providing hope for anti-plasmodial drugs (Mishra et al., 2017; Ward et al., 2022).

One approach to identifying novel therapies involves the optimization of existing treatments. This process involves the concurrent use of two or more drugs with distinct molecular targets or mechanisms of action. These therapies, which are either artemisinin-based or non-artemisinin-based combinations, are designed to mitigate potential toxicities and prevent recurrence and possible emergence of clinical resistance (Mishra et al., 2017; Kavishe et al., 2017; Eduardo de Souza et al., 2019). Therefore, combinations of antiplasmodial compounds are proving to be a way forward for developing more effective therapies. Moreover, the complexity of the factors involved in malaria pathogenesis underscores the need for multi-target therapeutic strategies (Tarkang et al., 2016). Thus, identifying new antiplasmodial combinations or compounds with multitarget actions would significantly reduce malaria-associated morbidity.

In regions where malaria is prevalent, impoverished communities often depend on local medicinal plants for treatment because of their cost-effectiveness and ease of access compared with standard pharmaceuticals (Adia et al., 2016). Despite this reliance, scientific validation of the antiparasitic effects of these plants is scarce. *Lepidobotrys staudtii*, a plant from the family *Lepidobotryaceae*, is predominantly found in southern and eastern Cameroon, where its bark is used in decoctions or macerations to alleviate malarial fevers. Studies have indicated that compounds isolated from this plant (Table S1) can inhibit cellular DNA polymerases and reverse transcriptases of HIV-1 and HIV-2 (Bokesch et al., 1996). In addition, decoctions made from plant roots are utilized as diuretics and possess ophthalmic properties (Sillans, 1951). To date, no research has been conducted to demonstrate the antiplasmodial properties of *L. staudtii* or any other plants in the same family. Given that *L.* *staudtii* is used by locals to treat burns and malarial fevers (Tane et al., 1996), we hypothesized that the plant extracts possess antiplasmodial activity. This study aimed to explore the blood-stage antiplasmodial activities of *Lepidobotrys staudtii* stem bark against chloroquine-sensitive (*Pf3D7*) and chloroquine-resistant (*PfINDO)* and multi-drug resistant *(PfDd2)* of human malarial parasites.

## 2 Materials and methods

### 2.1 Materials

RPMI 1640+GlutaMAX ™ -1 (Ref: 61870-036, Gibco, United states), AlbuMAX de type I, Ref: 110020-039, Gibco, New Zealand); Hypoxanthine-thymidine (Ref: 11067-030, Gibco, United states); Gentamycin (Ref: 15750-060, Gibco, China); Chloroquine diphosphate salt (Ref: C6628, Sigma); Giemsa R solution (Ref: 32884, RAL diagnostics); D-Sorbitol (Ref: 85529, Sigma); Saponin (Ref: 47036, Sigma); Triton X-100 (Ref: A16046, Alfa Aesar); HEPES buffer 1M (Ref: 11560496, Gibco); Phosphate buffer Saline (Ref: 10010-023, Gibco); Trypan Blue stain 0.4 % (Ref: 15250-061, Gibco); Trypsin-EDTA solution 10X (Ref: T4174, Sigma); SyBr Green I 10000X (Ref: S7585, Life technologies); Glucose solution 200 g/L (Ref: A24940-01, Gibco); Artemisinin (Ref: 361593, Sigma); Methanol (Ref: 20834.29, VWR Chemicals); DMSO (Ref: 10127403, Thermoscientific); Chloride sodium (Ref: 27810.262, AnalR NORMAPUR); histopaque-1077 (Ref: 10771, Sigma); DCF-DA (Ref: D6883, Sigma). *Pf* 3D7(MRA-102), *Pf* Dd2 (MRA-156), and *Pf* INDO were obtained from Biodefense and Emerging Infections (BEI) Research Resources (Manassas, VA, USA). The CyQUANT XTT Cell Viability Assay Kit was obtained from Invitrogen. Ascorbic acid, Tris-base, dihydroartemisinin (Sigma), lumefantrine (Sigma), and phytochemical screening reagents were of analytical grade.

### 2.2 *Lepidobotrys staudtii* crude extract and fractions preparation

The stem bark of the plant was harvested in Mintom (Eastern Cameroon) in January 2022 and identified by Mr. Sonke (Botanist) at the National Herbarium under voucher specimen N° 65847/HNC.

**Fig. 1:**
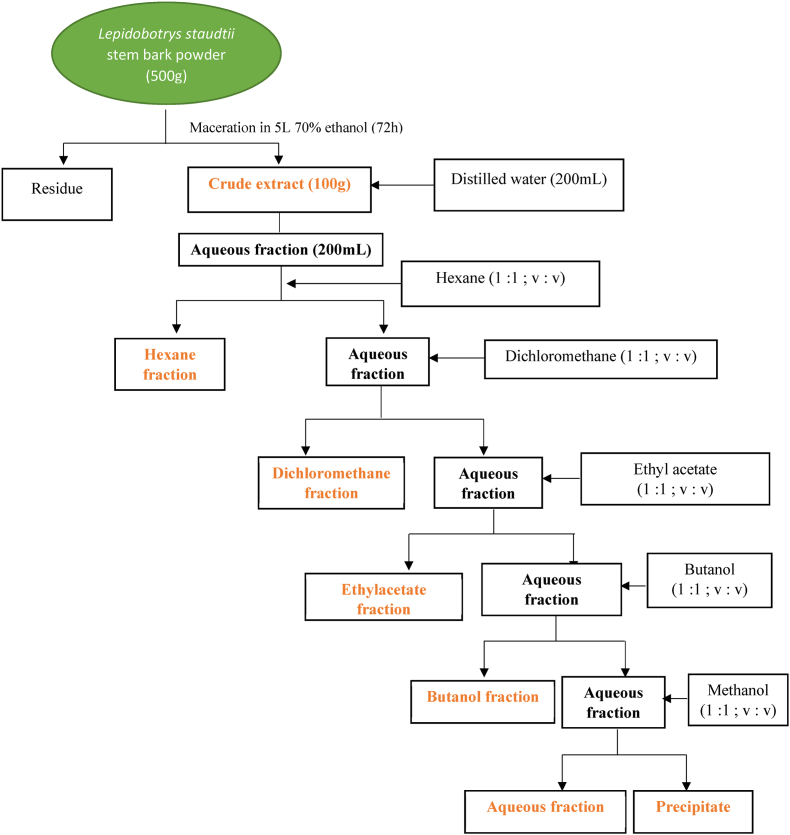
Flow chart of the extraction and fractionation of *Lepidobotrys staudtii* stem bark.

The plant material obtained was adequately cleaned, cut into small pieces, air-dried, and ground to obtain a powder. The dried powder (1000 g) was macerated and constantly stirred in 5 L 70 % ethanol for 72 h. The mixture was then filtered and oven-dried at 55 °C for 9-day period to obtain a dry brown paste (170 g, 17 % yield). The obtained crude extract was fractionated by the liquid-liquid partition method (volume: volume) using solvents of increasing polarity (hexane, dichloromethane, ethyl acetate, and butanol) according to the protocol described (Mfopa et al., 2017). Briefly, 100 g of the crude extract was dissolved in 200 mL of distilled water (aqueous fraction) and transferred to a 500 mL funnel. Two hundred milliliters of hexane were then added to the funnel and the mixture was shaken vigorously for 3 min and left on a stand for 5 min to allow separation. The aqueous fraction and the upper fraction (hexane fraction) were then collected separately. The lower aqueous fraction was then mixed with an equal volume of hexane and extracted repeatedly until clear hexane was collected. To ensure the total extraction of compounds soluble in hexane, thin-layer chromatography was performed before changing the solvent in an increasing polarity manner (Fig. 1). After recovering the butanol fraction and adding methanol to the residue for water evaporation, a precipitate was formed in the separating funnel, in addition to the aqueous fraction. The obtained fractions were concentrated using a rotavapor, air-dried until the solvent was completely evaporated, and the respective residues were screened for *in vitro* antiplasmodial and cytotoxic activity.

### 2.3 *In vitro* culture of *Plasmodium falciparum* strains

Parasite strains of *Pf*3D7 (sensitive to chloroquine) and *Pf*INDO (resistant to chloroquine) were maintained in human erythrocyte culture at 4–5 % hematocrit in RPMI 1640 medium supplemented with 5 g/L AlbuMAX (lipid-rich bovine albumin), 2 g/L glucose, 75 mg/L hypoxanthine, 2 g/L sodium bicarbonate, and 10 mg/L gentamycin sulfate and incubated at 37 °C in a low-oxygen environment using the Candle jar method (Trager and Jensen, 1976). *Pf*Dd2 (resistant to chloroquine, mefloquine, pyrimethamine and sulfadoxine) was maintained in RPMI culture medium containing GlutaMAX and NaHCO_3_, supplemented with 25 mM HEPES, 1X hypoxanthine, 40 μg/mL gentamicin, and 0.5 % AlbuMAX I at 37 °C in a CO_2_ incubator using fresh human blood at 3 % hematocrit, as described by Trager and Jensen (1976).

### 2.4 Erythrocytes preparation

Erythrocytes were obtained from fresh human blood under sterile conditions, as described previously (Radfar et al., 2009), with some modifications. Blood was collected in a 10 mL syringe and transferred into falcon tubes containing 1 mL of Citrate-Phosphate-Dextrose-adenine (CPDA), or blood bags centrifuged at 4000 rpm for 10 min. The plasma was discarded, and the pellet was mixed with an equal volume of Histopaque and centrifuged to completely eliminate peripheral blood mononuclear cells (PBMC). This step was followed by a series of two washes (4000 rpm, 10 min) with incomplete RPMI medium. After the last wash, erythrocytes with 50 % hematocrit were prepared by adding an equal volume of incomplete RPMI to that of the final erythrocyte pellet and stored at +4 °C.

### 2.5 Drugs and compounds dilution

Stock solutions of crude extract, fractions, lumefantrine (LUM), dihydroartemisinin (DHA), and DCF-DA were dissolved in 100 % DMSO, whereas chloroquine (CQ) was dissolved in sterile distilled water at 10 mg/mL concentration. Prior to experimentation, the stocks were diluted in complete RPMI medium to obtain the desired concentration.

### 2.6 Growth inhibition assay

The *in vitro* antiplasmodial activities of asexual blood-stage parasites were assessed in asynchronous cultures (mixed stages) of *Pf*3D7 and *Pf*INDO strains, whereas synchronized ring cultures were used for the *Pf*Dd2 strain. Antiplasmodial activity was demonstrated by the SYBR-green I fluorescence assay, as described (Smilkstein et al., 2004), with slight modifications. SYBR green I is a fluorescent compound that quantifies parasite DNA by emitting fluorescence following its intercalation between DNA double strands. It is a reliable test for demonstrating antiplasmodial activity because, given the absence of DNA in erythrocytes, the observed fluorescence represents only parasite DNA (Grimberg, 2011).

Briefly, all samples were 2-fold serially diluted in duplicate with the complete culture medium to reach final concentrations ranging from 25 to 0.78 μg/mL (<0.5 % DMSO) in 96-well microdilution plates. The samples were exposed to asynchronous *Pf*3D7 and *Pf*INDO cultures with 2 % hematocrit and 1 % parasitemia. Chloroquine (1000 μg/mL for *Pf*3D7 and 2000 μg/mL for *Pf*INDO) and Dihydro-artemisinin (10 μg/mL for *Pf*3D7 and 20 μg/mL for *Pf* INDO) was used as the positive control to achieve complete parasite inhibition. Complete medium without the test molecules was used as the negative control (maximal growth). After incubation, the plates were exposed to100 μL of SYBR Green I solution (0.03 μL/mL, final concentration) prepared in lysis buffer (20 mM Tris (pH 7.5), 5 mM EDTA, 0.008 %; w/v saponin and 0.08 %; v/v Triton X-100), gently mixed by pipetting, and incubated in the dark for 2h for parasite DNA intercalation. Fluorescence values were measured using a multi-mode plate reader at excitation and emission wavelengths of 485 nm and 528 nm, respectively (Synergy Bioteck). Analysis were performed using Microsoft Excel software as described by (Rajendran and Naveen, 2024). Percent parasite viabilities were calculated from fluorescence data using formula (1) below: Sigmoidal dose-response curves were derived by plotting percent viabilities against drugs concentration. Half maximal inhibitory concentrations values (IC_50_ values) were determined by projection onto dose-response curves. Each IC_50_ value represents the mean ± standard deviation (SD) of three independent experiments.

(1) $$\%Viability=\frac{T-\mathrm{PC}}{D}\times 100$$

Where:

T: Mean fluorescence (Test)

PC: Positive control.

D: Difference between the negative and positive controls.

For *Pf*Dd2, parasite growth inhibition was assessed as described by (Efange et al., 2022). A synchronized ring-stage culture with 5 % sorbitol treatment was maintained for one cycle before biological activity testing. Two-fold serially diluted samples were co-cultured with parasites (1 % parasitemia and 1.5 % hematocrit) for 72h in 96-well plates to final drug concentrations ranging from 100 to 0.78 μg/mL (0.2 % DMSO, final concentration) in a final volume of 100 μL. Artemisinin (1 μM) was used as a positive control, whereas solvent-treated culture (0.2 % DMSO) was used as a negative control. Mean half-maximal inhibitory concentrations (IC_50_ values) were calculated by plotting percentage growth inhibition versus log drug concentration and fitting response data to a variable slope sigmoidal curve fitting function using Graph Pad Prism v8.0. The IC_50_ values represent the mean ± standard deviation of three independent tests.

The antiplasmodial activities where categorized based on WHO guidelines and Jonville and collaborators where crude extract and fractions activities are classified as: highly active (IC_50_ < 5 μg/mL), promisingly active (5≤ IC_50_ < 15 μg/mL), moderately active (15≤ IC_50_ < 50) and inactive (IC_50_ ≥ 50 μg/mL) (Jonville et al., 2008).

### 2.7 *In vitro* measurement of cytotoxic activity against mammalian cell lines

The cytotoxic effects of the crude extract and its fractions were determined in Normal African Green Monkey Kidney Epithelial cells (Vero) using an XTT viability assay. The cells were maintained in DMEM supplemented with 10 % FBS, 20 mM HEPES, and 1 % penicillin-streptomycin. Confluent cells were trypsinized, seeded at a density of 100, 000 cells/wells (total volume 90 μL) in 96-well plates, and incubated for 24 h prior to drug treatment. Two-fold serial dilutions of extract and fractions (500-3.9 μg/mL) were added to the plates (10 μL) and incubated under humidified conditions at 37 °C for 72h. Cell viability was assessed using the XTT cell viability assay kit according to the manufacturer's instructions. The absorbance of the formazan product was measured at 450 nm using a MULTISKAN FC microtiter plate reader. The 50 % cytotoxic concentration (CC_50_) was obtained from dose-response curves using GraphPad Prism software version 8.0 for class of toxicity determination according to (Malebo et al., 2010). Based on cytotoxicity studies, a molecule, an extract or a fraction can be categorized as highly toxic (CC_50_ < 1 μg/mL), moderately toxic (1<CC_50_ < 10 μg/mL), midly toxic (1<CC_50_ < 30 μg/mL) and non-toxic (CC_50_ > 30 μg/mL) (Malebo et al., 2010). The relative toxicity of the observed antiplasmodial activity was assessed by calculating the selectivity index (SI = CC_50_/IC_50_) (Efange et al., 2022).

### 2.8 *In vitro* assessment of hemolytic activity against uninfected human erythrocytes

The hemolytic effect of the crude extract and fractions on normal human erythrocytes was assessed spectrophotometrically by measuring the amount of released haemoglobin into the culture medium, as reported by (Rajendran et al., 2018) with slight modifications. One hundred microliters of fresh human erythrocytes diluted in incomplete RPMI medium (4 % hematocrit) were added to 100 μL of diluted fractions (500–0.97 μg/mL) and incubated for 48h at 37 °C. The absorbance of the supernatant was measured at 450 nm wavelength. Distilled water was added 2 h prior to the end of the incubation period and served as a positive control, and 0.5 % DMSO in complete media served as the negative control. Percent hemolysis was calculated using the following formula: percentage hemolysis = (absorbance value of test sample – absorbance value of negative control/absorbance value of positive control) × 100. Percent hemolysis represents the mean ± standard deviation of three independent tests, each in duplicate.

### 2.9 Time-kinetic growth inhibition assay on *P. falciparum*

The time-kinetic action of the crude extract and fractions against *Pf*3D7 and *Pf*INDO was determined as described by (Rajendran and Naveen, 2024). Briefly, one-hundred microliter of asynchronous parasite culture (2 % hematocrit and 1 % parasitemia) were added to two-fold serially diluted fractions (25–0.78 μg/mL) in a 96 wells plate and incubated for 12, 24 and 96h. For the 96h incubation, parasites were added at 0.5 % parasitemia. After each incubation time, the culture plates were exposed to SYBR Green I solution and fluorescence was measured at excitation and emission wavelengths of 485 nm and 528 nm, respectively. The fluorescence readings were used to determine percent parasite viability. The 50 % inhibitory concentrations of the test compounds were determined by plotting the percent viability versus drug concentration.

### 2.10 *In vitro* drug interaction with standard antimalarials

Drug interactions between *Lepidobotrys staudtii* fractions and standard antimalarial drugs chloroquine, lumefantrine, and/or dihydro-artemisinin were studied against *Plasmodium falciparum* 3D7 and INDO as described by (Eid et al., 2012a) with slight modifications. The fractions IC_50_ in the presence of standard drugs at a fixed concentration lower than their IC_50_ was determined. The fractions were 2-fold serially diluted (25–0.78 μg/mL) in a final volume of 50 μL followed by the addition of 50 μL of chloroquine (final concentrations of 10 ng/mL for *Pf* 3D7 or 50 ng/mL for *Pf* INDO) or DHA (final concentrations of 3 ng/mL and 1.5 ng/mL for both strains) or lumefantrine (final concentrations of 10 ng/mL for *Pf* 3D7 or 14 ng/mL for *Pf* INDO) to each well. Then, 100 μL of parasite culture (2 % hematocrit, 1 % parasitemia) was added to each well, and the plates were incubated at 37 °C in a low-oxygen environment for 48h, after which parasite viability was determined using a SYBR Green I assay, as previously described. The nature of the interactions between the fractions and the reference drugs was assessed using the combination index method as described by (Eid et al., 2012a) following formula (2) below, where interactions are classified as synergistic (CI < 1), additive (CI = 1) or antagonistic (CI > 1). In order to see if the combinations affected fractions activity against Plasmodial strains, we determine the reversal ratio (RR) by doing the ratio between IC_50_ of fractions alone and IC_50_ of fractions in combination. Interactions were considered as beneficial for RR > 1.

(2) $$CI=\frac{{IC}_{50\;}in\;combination}{{IC}_{50\;}alone\;}+\frac{Fixed\;concentration\;of\;reference\;drug}{{IC}_{50\;}of\;reference\;drug}$$

Additionally, a graphic representation of the interactions between fractions and drugs was made using the isobologram approach. The IC_50_ concentrations of the fractions and reference drugs were plotted on the X-and Y axis in a two-coordinate plot, corresponding to (0, X_A_) and (0, X_B_), respectively. The X_A_ and X_B_ concentrations of the two partners’ drugs used in combination to provide the same effect were placed in the same plot. Synergy, additivity and antagonism were observed for X_A_ and X_B_ located below the line, on the line and above the line, respectively.

### 2.11 Blood stage specific activity of *Lepidobotrys staudtii* crude extract and fractions on *Pf*3D7 and *Pf*INDO

To determine the stage-specific activity of the fractions, parasite cultures were synchronized using 5 % D-sorbitol for 10 min with slight modifications. At ring, trophozoite or schizont stages, parasites were exposed to 2-fold serially diluted fractions (25–0.78 μg/mL) in a 96-well plate and incubated for 24h. The respective IC_50_ values were determined using the SYBR Green I assay as previously described.

### 2.12 Effect of fraction on reactive oxygen species generation against *Pf*INDO

The effect of the crude extract and fractions on the reactive oxygen species generation was assessed on *Plasmodium falciparum* INDO using dichlorofluorescein diacetate (DCF-DA), an assay based on the oxidation of DCFH to a highly fluorescent compound (DCF). Briefly, 5 % sorbitol synchronized cultures were treated at the trophozoite stage (final hematocrit 2 % and 1 % parasitemia) with DCF-DA (final concentrations 80 and 40 μM) and incubated in the dark at 37 °C for 30 min. After incubation, the cultures were washed twice using incomplete media and incubated for 6 h with diluted fractions (25-12.5 μg/mL), artemisinin (100-50 ng/mL) or ascorbic acid (80-40 μM). DCF fluorescence was measured at the excitation and emission wavelengths of 485 and 528 nm, respectively.

### 2.13 Phenotypic assay

The phenotypic assay was carried out with the most promising EA fraction on *Pf*INDO following the protocol described by (Efange et al., 2022), with some modifications. Briefly, a culture containing most parasites at the ring stage was tightly synchronized by 5 % D-sorbitol treatment. The culture was diluted (1 % parasitemia, 2 % hematocrit), and at the mid-ring (∼10h post invasion), early trophozoites (∼20h post invasion), and mid-schizonts (∼40h post invasion), the fraction was added in duplicate at final concentrations of 10 and 20 μg/mL (∼2xIC_50_ and ∼4xIC_50_) and incubated at 37 °C for 24h. After exposure, parasite progression was monitored at time intervals of 12h for any developmental growth arrest for ring or trophozoite stages up to 24h by Giemsa-stained thin blood smear under 100x light microscopy. Schizont stage maturation was monitored every 4h using Giemsa-stained blood smears. The morphological defects at each stage at the respective time points were captured under a microscope using the Magvision software.

### 2.14 Phytochemical constituents of *Lepidobotrys staudtii* plant extracts and fractions

Phytochemical screening of the crude extract and fractions was performed as described by (Yadav and Agarwala, 2011) (Table S2). The total phenolic and flavonoid content was analyzed according to the protocols described in (Ngoungoure et al., 2019), with slight modifications.

#### 2.14.1 Total phenol and flavonoid contents

The total phenol content (TPC) was determined using the Folin-Ciocalteu reagent (FCR) method, with gallic acid (GA) as the phenolic standard. The calibration curve was plotted using six different dilutions of GA ranging from 10 to 100 μg/mL. One milliliter of each GA dilution and sample (initial concentration of 1 mg/mL) was mixed with 5 mL FCR and 4 mL of 7.5 % sodium carbonate. The reaction mixture was then incubated in the dark at room temperature for 45 min. The absorbance of the mixture was measured at 750 nm using a Shimadzu UV–visible spectrometer. The TPC of the samples was expressed in milligrams of gallic acid equivalents per gram of extract (mg GAE/g extract or fraction). TPC values represent the mean ± standard deviation of two independent tests, each performed in triplicates.

Total flavonoid content (TFC) was determined using an aluminum chloride (AlCl_3_) colorimetric assay. Quercetin was used as a standard compound to determine the total flavonoid content in the plant samples. The calibration curve was plotted using six different dilutions of quercetin ranging from 10 to 100 μg/mL. Four milliliters of each standard dilution and sample were added to a 10 mL standard flask containing 0.2 mL of saturated potassium acetate. After a few minutes 0.2 mL of 2 % AlCl_3_ and 5.6 mL and distilled water was added. The reaction mixture was then incubated in the dark at room temperature for 60 min. Absorbance was measured at 436 nm using a Shimadzu UV–visible spectrometer. The TFC of the samples was expressed as mg of quercetin equivalent (QE)/g of the extract or fraction. The TFC values represent the mean ± standard deviation of two independent tests, in which each sample was analyzed in triplicate.

### 2.15 Reverse Phase High Performance Liquid Chromatography (RP-HPLC)

Fractions were subjected to analytical RP-HPLC using Shimadzu RP-HPLC (model: i – series LC- 2050C) fitted with a C_18_ column (250 mm × 4.6 mm, 5 μm particle size). The efficient chromatographic separation is achieved by the isocratic low pressure gradient elution with mobile phase comprising of Methanol and water in the ratio (80:20 v/v). Both the Methanol and water were degassed for 30 min using an ultrasonic bath sonicator. The samples were prepared by dissolving 1 g of crude extract or each fraction in 10 mL of HPLC-methanol grade and sonicating for 15 min at 40 °C to obtain a final concentration of 100 mg/mL. After sonication, the methanolic extract was filtered using a 0.22 μM PTFE syringe filter to remove any particles. The diluted extracts were then diluted to a concentration of 1Kppm using pure methanol. For each injection, the volume was 10 μL and the flow rate was set at 1 mL/min.

### 2.16 Statistical analysis

The results are presented as mean ± standard deviation of at least two independent experiments, each performed in duplicate. Fifty percent inhibitory concentrations (IC_50_s) were determined by dose-response curve analysis, either by non-linear regression using GraphPad Prism Version 8.0 (*Pf* Dd2), or by determination of viability percentages using Excel 2016 (*Pf* 3D7 and *Pf* INDO). Analyses were performed using one-way analysis of variance (ANNOVA and unpaired *t*-test, with statistical significance set at *p* ≤ 0.05. For stage specificity, the IC_50_ values obtained for each stage were compared with those obtained from growth inhibition tests. For time kinetics, the IC_50_ values obtained at each incubation time were compared, whereas for the phenotypic test, the treatment was compared with the control. For the combination studies, the IC_50_ in combination was compared to IC_50_ alone.

## 3 Results

### 3.1 Crude extract and fractions preparation

The powder maceration yielded to 170 g of hydro ethanol crude extract, from which 100g was subjected to liquid-liquid partition using solvents of increasing polarity. This resulted in hexane (HXN), dichloromethane (DCM), ethyl acetate (EA), butanol (BOH), aqueous (AQ), and a precipitate (PPX). Fractionation yields revealed that the BOH and EA fractions were the largest with yields of 34.1 and 10.53 % respectively, followed by AQ (8.3 %), DCM (6.34 %), HXN (3.1 %) and PPX (2.79 %).

### 3.2 *In vitro* antiplasmodial and cytotoxic activities of *L. staudtii* crude extract and fractions against *P. falciparum* strains

The antiplasmodial activities of both the crude extract and fractions were assessed against *Pf*3D7, *Pf*Dd2, and *Pf*INDO strains using SYBR Green I assay after 48h of exposure in asynchronous or synchronous cultures. The antiplasmodial activities of the crude extract and fractions are presented in Table 1. The crude extract exhibited high to promising antiplasmodial activities with IC_50_ values of 5, 7.18 and 2.02 μg/mL against *Pf*3D7, *Pf*INDO, and *Pf*Dd2, respectively. Also, tested fractions (HXN, DCM, EA, BOH, AQ and PPX) exhibited promising antiplasmodial activities with IC_50_ values ranging from 3.73 to 7.13 μg/mL for *Pf*3D7, from 4.8 to 7.85 μg/mL for *Pf*INDO and from 1.07 to 5.58 μg/mL for *Pf*Dd2 strain. As experimental controls, standard antimalarial drugs, such as CQ, ART, and DHA, showed IC_50_ values at sub-nanomolar concentrations against sensitive and resistant strains (Table 1). Furthermore, in order to determine potential cross-resistance, resistance indices (RI) were determined by calculating the ratio between IC_50_ values obtained against the resistant strains and those obtained against the sensitive strain. The resistance indices were found to be less than 2.

**Table 1:** Parasite viability, cytotoxicity, resistance index and selectivity index of *Lepidobotrys staudtii* crude extract and fractions.

Fractions	IC_50_ (μg/mL)			Resistance Index		CC_50_ on Vero cells (μg/mL)	SI
	*Pf* 3D7	*Pf* INDO	*Pf* Dd2	IC_50_ INDO/IC_50_ 3D7	IC_50_ Dd2/IC_50_ 3D7		
**CE**	5.0 ± 1.00	7.23 ± 0.82	2.02 ± 0.59	1.44	0.34	>500	>100
**HXN**	3.73 ± 1.1	7.25 ± 2.2	5.51 ± 2.57	1.95	1.2	115.6	30.99
**DCM**	4.3 ± 1.28	5.93 ± 1.67	7.33 ± 2.82	1.38	1.3	314.3	73.09
**EA**	3.9 ± 0.92	4.8 ± 0.52	1.62 ± 0.24	1.23	0.41	>500	>128.2
**BOH**	5 ± 0.2	6.6 ± 1.7	1.7 ± 0.58	1.32	0.21	>500	>100
**AQ**	7.13 ± 0.47	7.5 ± 1.03	3.78 ± 1.29	1.05	0.63	>500	>70.12
**PPX**	6.63 ± 1.32	7.85 ± 2.00	3.37 ± 0.47	1.18	0.61	>500	>75.41
**CQ**	0.015 ± 2.15	0.22 ± 21.92	0.04 ± 13.97	16.57	2.95	ND	ND
**ART**	0.003 ± 1.3	0.034 ± 7.35	0.005 ± 1.07	11.33	1.66	ND	ND
**DHA**	0.007 ± 2.59	0.0031 ± 0.96	ND	0.4	ND	ND	ND
**LUM**	0.017 ± 2.97	0.028 ± 8.08	ND	1.64	ND	ND	ND

*In vitro* blood-stage antiplasmodial activity and cytotoxicity of *Lepidobotrys staudtii* crude extract and fractions against *P. falciparum* strains and Vero cells. Results are expressed as mean ± SD of at least three independent experiments each in duplicate. CE: *Lepidobotrys staudtii* crude extract; HXN: hexane fraction; DCM: dichloromethane fraction; EA: ethyl acetate fraction; BOH: butanol fraction; AQ: aqueous fraction; PPX: precipitate; CQ: chloroquine; DHA: dihydro-artemisinin; ART: artemisinin; LUM: lumefantrine; ND: Not determined. SI: selectivity index = CC_50_ on Vero cells/IC_50_*Pf* 3D7.

Next, we determined the effect of the extract and fractions on Vero cell viability by determining the 50 % cytotoxic concentrations (CC_50_). CE, EA, BOH, AQ, and PPX showed CC_50_ values > 500 μg/mL followed by DCM (314.3 μg/mL) and HXN (115.6 μg/mL). This indicated that the fractions induced cell toxicity at concentrations of 50–100 times relative to the antiplasmodial IC_50_ values. We also determined the selectivity index (SI) based on the CC_50_ of Vero cells upon IC_50_ of *Pf*3D7 or *Pf*INDO, and the results indicated that all SI values were greater than 10 (Table 1). The hemolytic activity of the crude extract and fractions was determined spectrophotometrically. Fractions were tested at concentrations varying between 500 and 0.97 μg/mL with 4 % hemotocrit for 48h and lysis percentages were determined. In general, the extracts showed a low percentage of RBC cells. Indeed, apart from the HXN fraction, which recorded a lysis percentage of 79.48 % at 500 μg/ml, all other extracts recorded percentages varying between 13.96 and 34.19 %. Moreover, for concentrations close to the IC_50_ values obtained (7.81–0.97 μg/ml) against plasmodial strains, the lysis percentages were relatively low, varying between 1.75 and 0 % (Table 2).

**Table 2:** Hemolytic percentages of crude extract and fractions from *L. staudtii* stem bark in human erythrocytes.

Fractions	%Hemolysis									
	Samples tested concentrations (μg/mL)									
	500	250	125	62.5	31.25	15.63	7.81	3.9	1.95	0.97
**CE**	20.72 ± 7.05	8.99 ± 0.02	5.11 ± 0.58	2.81 ± 0.23	1.79 ± 0.32	1.01 ± 0.29	0.62 ± 0.38	0.81 ± 0.23	0.47 ± 0.29	0.07 ± 0.06
**HXN**	79.48 ± 7.12	3.86 ± 0.34	0.11 ± 0.23	0.2 ± 0.00	1.68 ± 2.96	0.41 ± 0.99	0.15 ± 0.68	0.02 ± 0.51	0.0 ± 0.25	0.0 ± 0.68
**DCM**	13.96 ± 1.37	5.41 ± 1.94	2.35 ± 0.64	0.94 ± 0.24	0.44 ± 0.28	0.29 ± 0.37	0.00 ± 0.65	0.08 ± 0.36	0.0 ± 0.41	0.0 ± 0.48
**EA**	34.19 ± 2.94	15.66 ± 1.8	7.58 ± 0.75	4.03 ± 0.48	2.32 ± 0.48	1.45 ± 0.58	0.77 ± 0.42	0.58 ± 0.02	0.17 ± 0.48	0.03 ± 0.61
**BOH**	20.98 ± 1.34	9.45 ± 0.87	5.09 ± 0.61	2.84 ± 0.24	1.6 ± 0.19	1.23 ± 0.02	0.78 ± 0.14	0.44 ± 0.77	0.76 ± 1.27	0.67 ± 0.53
**AQ**	19.7 ± 1.38	9.72 ± 1.83	5.16 ± 1.48	3.21 ± 0.55	1.95 ± 0.3	1.38 ± 0.23	1.00 ± 0.63	0.7 ± 1.4	0.42 ± 0.37	0.18 ± 0.04
**PPX**	20.85 ± 3.33	11.67 ± 3.4	5.28 ± 1.58	3.63 ± 1.08	1.86 ± 0.34	1.13 ± 0.34	1.75 ± 1.57	1.27 ± 1.35	0.79 ± 0.09	0.21 ± 0.13

Hemolytic effects of *L. staudtii* crude extract and fractions on fresh human erythrocytes. Serially diluted fractions were incubated with fresh human erythrocytes for 48 h at 2 % hematocrit, after which the absorbance of the supernatant was measured at 450 nm. Results are expressed as mean ± SD of three independent experiments each done in duplicate. CE: *Lepidobotrys staudtii* crude extract; HXN: hexane fraction; DCM: dichloromethane fraction; EA: ethyl acetate fraction; BOH: butanol fraction; AQ: aqueous fraction; PPX: precipitate.

For further work, due to the non-availability of the *Pf*Dd2 strain, tests were carried out only on the *Pf*3D7 and *Pf*INDO strains.

### 3.3 *In vitro* speed of action of plants fractions

The speed of action is a key factor in the antimalarial efficacy. To determine whether crude extract and fractions are fast- or slow-acting agents, 2-fold serially diluted fractions and crude extract were exposed to asynchronous *Pf*3D7 and *Pf*INDO parasite cultures for 12 h, 24 h, and 96 h.

**Fig. 2:**
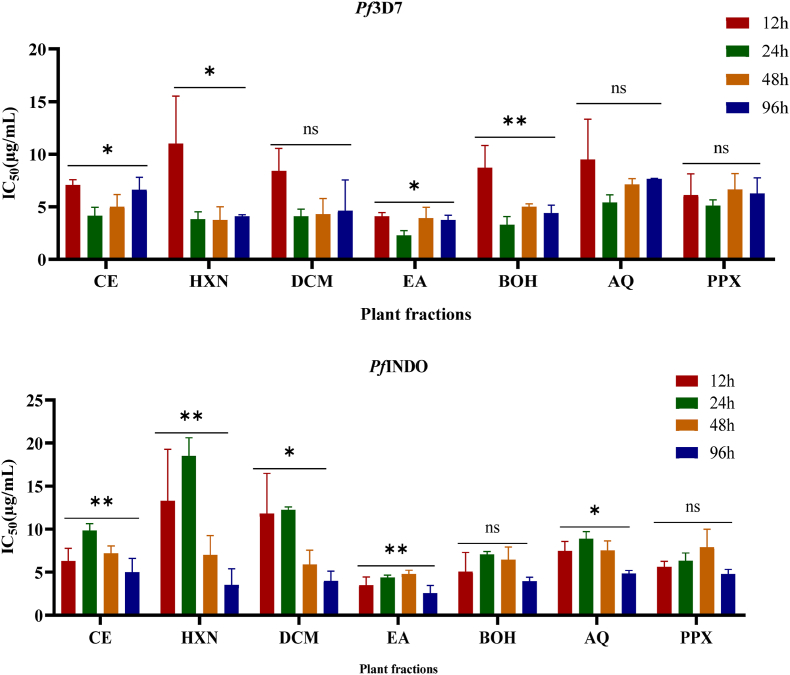
Time-kinetic inhibition assay. Asynchronous *P. falciparum* cultures were treated with the fractions for different time-points, and parasite viability was evaluated using the SYBR Green assay determining their IC_50_ values. The findings are presented as the mean ± SD from at least three separate experiments, each conducted in duplicate. CE: Lepidobotrys staudtii crude extract; HXN: hexane fraction; DCM: dichloromethane fraction; EA: ethyl acetate fraction; BOH: butanol fraction; AQ: aqueous fraction; PPX: precipitate. ∗ Indicates significant differences between various time points (*p* < 0.05).

In the *Pf*3D7 strain, after 12 h of exposure, only the EA fraction had a high activity with an IC_50_ value of 4.07 μg/ml. Further prolonged exposures for 24, 48, and 96 h showed significant reduction in IC_50_ values of CE, HNX, DCM, EA, and BOH fractions, compared to the 12 h exposure (Fig. 2, S2). Thus, CE, HXN, DCM, EA, and BOH fractions were moderate fast-acting agents, as they started to show good activity after 24 h of treatment, and this activity was maintained for up to 96 h of treatment. However, the fastest-acting fraction was EA, which showed promising activity after 12 h of treatment, with IC_50_ values of 4.05 μg/mL. Apart from the DCM, BOH, and PPX fractions, there was a significant difference (*p < 0.05*) in the activities of the other fractions according to the exposure time. Against *Pf* INDO, after 12 h, 24 h and 48 h of exposure, only the EA fraction showed promising antiplasmodial activity with respective IC_50_ values of 3.5, 4.4 and 4.8 μg/mL. However, after 96 h of exposure, all the fractions showed promising activity (IC_50_ values ranging from 2.6 to 4.85 μg/mL) (Fig. 2, S2). Accordingly, we can assert that the HXN, DCM, AQ, and PPX fractions display a delayed response against the *Pf*INDO strain, whereas the EA fractions exhibit dual behavior, comprising both fast and slow action.

This is evidenced by their promising activity levels across all exposure times, as shown in Fig. 2. Depending on the exposure time, there was a significant difference (*p* < 0.05) in the activities of the HXN, DCM, EA, and AQ fractions (Fig. 2).

### 3.4 Effect of fractions on *Plasmodium falciparum* erythrocytic stages

**Fig. 3:**
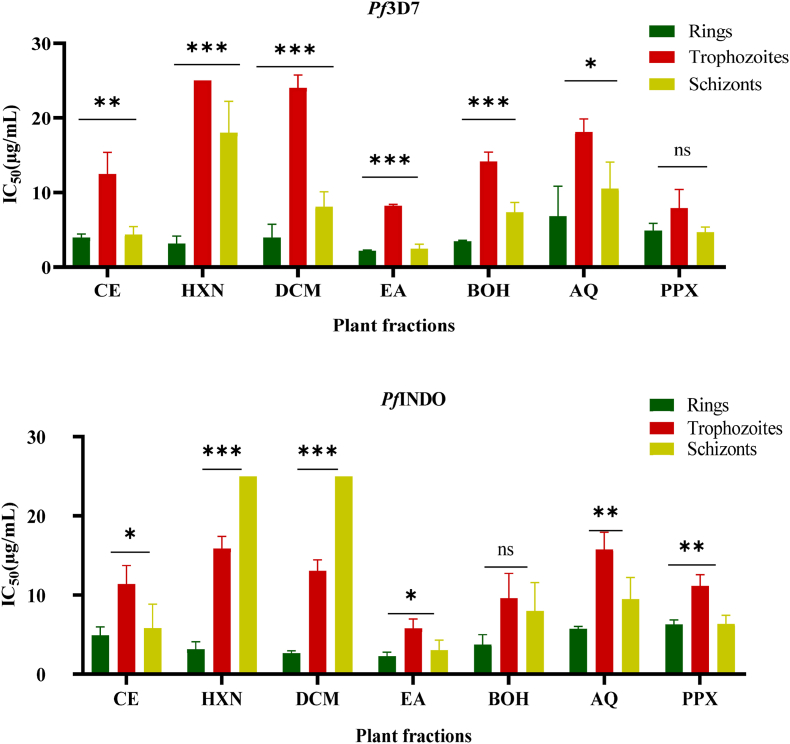
Stage-specificity assay. Synchronous cultures of *Pf* were treated with the fractions at various parasite stages (rings, trophozoites, or schizonts) for a duration of 24 h, and parasite viability was evaluated using the SYBR Green assay. The findings are presented as the mean ± SD from three independent experiments, each conducted in duplicate. CE: *Lepidobotrys staudtii* crude extract; HXN: hexane fraction; DCM: dichloromethane fraction; EA: ethyl acetate fraction; BOH: butanol fraction; AQ: aqueous fraction; PPX: precipitate. ∗: significant difference between ring, trophozoite, and schizont stages (*p* < 0.05).

Next, we determined the stage-specific antiplasmodial activity of the fractions against different developmental stages of *Pf*3D7 and *Pf*INDO. To achieve this, tightly synchronized parasite cultures were exposed to rings, trophozoites, or schizont stages to varying concentrations of the fractions and incubated for 24h. The results showed that fractions exhibited potent inhibitory activity against trophozoite development at low IC_50_ values in the range of 2.17 and 6.8 μg/mL for *Pf*3D7 and 2.27 and 6.27 μg/mL for *Pf*INDO (Fig. 3). In addition, fractions showed marked activity against the schizont-stage with IC_50_ values ranging from 2.47 to 20 μg/mL (*Pf*3D7) and 3.03–9.47 μg/mL (*Pf*INDO). However, against the trophozoite stage, only the EA fraction showed a promising activity with IC_50_ values of 8.2 μg/mL (*Pf*3D7) and 5.77 μg/mL (*Pf*INDO). For all the tested stages, the EA fraction exhibited the highest activity, with lower IC_50_ values. These findings suggest that the EA fraction is effective against ring, trophozoite, and schizont stages (Fig. 3).

### 3.5 Standard antimalarial drugs enhance the antiplasmodial activity of plants fractions

**Fig. 4:**
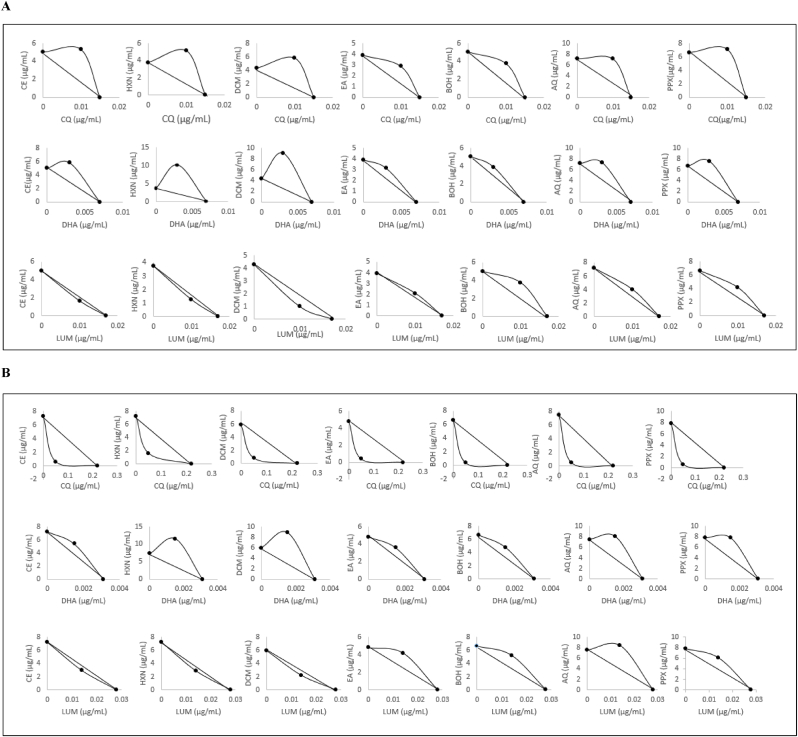
Isobologram analyses of combinations against *Pf*3D7 (A) and *Pf*INDO (B) strains: The IC_50_ concentrations of reference drugs are plotted on the x-axis and the IC_50_ values of the fractions on the y-axis. The line connecting these two points is the additivity line. Points below or above the line indicate synergy or antagonism respectively.

Based on their respective IC_50_ values, sub-inhibitory concentrations of chloroquine (CQ), dihydro-artemisinin (DHA), and lumefantrine (LUM) were selected, and their effects on fraction activities against *Pf*3D7 and *Pf*INDO strains were evaluated. The IC_50_ values of the fractions alone, in combination, the inversion ratios, the combination indices, and the isobolograms are shown in Table 3, Table 4, Table 5, and Fig. 4. Against *Pf*3D7, exposure of CQ or DHA (Table 3, Table 4) on fractions had an antagonistic effect with CI values varying between 1. 2 and 3.14, while combination with LUM had a synergistic effect with CE, HXN and DCM (CI values of 0.81 and 0.92) and an antagonistic effect with EA, BOH, AQ and PPX (CI values varying between 1.12 and 1.34) (Table 3, Table 4). Against *Pf*INDO, CQ had a synergistic effect with all the fractions (CI varying between 0.28 and 0.46) and IC_50_ values of fractions decreased from 4.38 to 18.43-fold (Table 3). In addition, there was a synergistic effect between LUM and the CE, HXN, and DCM fractions (CI: 0.87 and 0.91), while an antagonistic effect was observed with the EA, BOH, AQ, and PPX fractions (CI: 1.28–2.07) (Table 5). DHA had an antagonistic effect on all fractions (Table 4).

**Table 3:** *In vitro* antimalarial combination of fractions and chloroquine.

Fractions	*Pf* 3D7					*Pf* INDO				
	IC_50_ alone	IC_50_ in combination	RR	CI	Interaction	IC_50_ alone	IC_50_ in combination	RR	CI	Interaction
CE	5 ± 1.00	5.3 ± 1.82	0.94	1.73	Antagonism	7.23 ± 0.82	0.6 ± 0.1∗∗∗∗	12.05	0.31	Synergism
HXN	3.73 ± 1.1	5.1 ± 1.31	0.73	2.03	Antagonism	7.25 ± 2.2	1.6 ± 0.92∗∗	4.38	0.46	Synergism
DCM	4.3 ± 1.28	5.77 ± 0.21	0.75	2.01	Antagonism	5.93 ± 1.67	0.83 ± 0.31∗∗∗	7.08	0.37	Synergism
EA	3.9 ± 0.92	2.88 ± 0.73	1.36	1.40	Antagonism	4.8 ± 0.52	0.4 ± 0.00∗∗∗∗	12.00	0.31	Synergism
BOH	5 ± 0.2	3.72 ± 0.56	1.35	1.41	Antagonism	6.6 ± 1.7	0.35 ± 0.09∗∗	18.43	0.28	Synergism
AQ	7.13 ± 0.47	7.10 ± 0.98	1.00	1.66	Antagonism	7.5 ± 1.03	0.48 ± 0.03∗∗∗∗	15.62	0.29	Synergism
PPX	6.63 ± 1.32	7.11 ± 0.44	0.93	1.74	Antagonism	7.85 ± 2.00	0.6 ± 0.26∗∗∗	13.17	0.30	Synergism

Asynchronous cultures of *P.f* were incubated for 48h with serially diluted fractions combined with chloroquine at fixed sub inhibitory concentrations. Parasite viability was assessed using the SYBR Green test. Results are expressed as Means ± SD of at least three independent experiments each in duplicate. The IC_50_ values obtained were compared with the IC_50_ values of the fractions alone (*∗*: significant difference, *p < 0.05*). The reversal ratio and combination indexes were calculated. RR: Reversal Ratio; CE: *Lepidobotrys staudtii* crude extract; HXN: hexane fraction; DCM: dichloromethane fraction; EA: ethyl acetate fraction; BOH: butanol fraction; AQ: aqueous fraction; PPX: precipitate; CI: Combination Index (CI < 1: synergism; CI = 1: additivity; CI > 1: antagonism).

**Table 4:** *In vitro* antiplasmodial activity of fractions in combination with dihydro artemisinin.

Fractions	*Pf* 3D7					*Pf* INDO				
	IC_50_ alone	IC_50_ in combination	RR	CI	Interaction	IC_50_ alone	IC_50_ in combination	RR	CI	Interaction
CE	5 ± 1.00	5.8 ± 0.28	0.86	1.59	Antagonism	7.23 ± 0.82	5.35 ± 0.49∗	1.34	1.23	Antagonism
HXN	3.73 ± 1.1	10.1 ± 0.99∗∗	0.37	3.14	Antagonism	7.25 ± 2.2	11.5 ± 2.77	0.63	2.07	Antagonism
DCM	4.3 ± 1.28	9 ± 1.64∗	0.48	2.52	Antagonism	5.93 ± 1.67	8.8 ± 1.48	0.67	1.97	Antagonism
EA	3.9 ± 0.92	3.13 ± 0.99	1.24	1.23	Antagonism	4.8 ± 0.52	3.58 ± 0.64∗	1.34	1.23	Antagonism
BOH	5 ± 0.2	3.87 ± 0.9	1.29	1.20	Antagonism	6.6 ± 1.7	4.73 ± 0.71	1.40	1.20	Antagonism
AQ	7.13 ± 0.47	7.3 ± 1.67	0.98	1.45	Antagonism	7.5 ± 1.03	8.03 ± 0.66	0.93	1.55	Antagonism
PPX	6.63 ± 1.32	7.5 ± 0.98	0.88	1.56	Antagonism	7.85 ± 2.00	7.8 ± 0.5	1.01	1.48	Antagonism

Asynchronous cultures of *Plasmodium falciparum* were incubated for 48h with serially diluted fractions combined with dihydro artemisinine at fixed sub inhibitory concentrations. Parasite viability was assessed using the SYBR Green test. Results are expressed as Means ± SD of at least three independent experiments each in duplicate. The IC_50_ values obtained were compared with the IC_50_ values of the fractions alone (*∗*: significant difference, *p < 0.05*). The reversal ratio and combination indexes were calculated. RR: Reversal Ratio; CE: *Lepidobotrys staudtii* crude extract; HXN: hexane fraction; DCM: dichloromethane fraction; EA: ethyl acetate fraction; BOH: butanol fraction; AQ: aqueous fraction; PPX: precipitate; CI: Combination Index (CI < 1: synergism; CI = 1: additivity; CI > 1: antagonism).

**Table 5:** *In vitro* antimalarial combination of fractions and lumefantrine.

Fractions	*Pf* 3D7					*Pf* INDO				
	IC_50_ alone	IC_50_ in combination	RR	CI	Interaction	IC_50_ alone	IC_50_ in combination	RR	CI	Interaction
CE	5 ± 1.00	1.67 ± 0.64∗∗	3.00	0.92	Synergism	7.23 ± 0.82	2.93 ± 1.5∗∗	2.5	0.91	Synergism
HXN	3.73 ± 1.1	1.25 ± 0.44∗∗	2.98	0.92	Synergism	7.25 ± 2.2	2.87 ± 1.6∗	2.4	0.91	Synergism
DCM	4.3 ± 1.28	0.98 ± 0.46∗∗	4.41	0.81	Synergism	5.93 ± 1.67	2.2 ± 1.11∗	2.7	0.87	Synergism
EA	3.9 ± 0.92	2.08 ± 0.58∗	1.87	1.12	Antagonism	4.8 ± 0.52	4.2 ± 0.2	1.1	1.38	Antagonism
BOH	5 ± 0.2	3.77 ± 1.16	1.33	1.34	Antagonism	6.6 ± 1.7	5.23 ± 1.83	1.2	1.31	Antagonism
AQ	7.13 ± 0.47	4.00 ± 1.00∗∗	1.78	1.15	Antagonism	7.5 ± 1.03	8.37 ± 1.42	0.9	1.61	Antagonism
PPX	6.63 ± 1.32	4.17 ± 1.39	1.59	1.22	Antagonism	7.85 ± 2.00	6.13 ± 0.9	1.3	1.28	Antagonism

Asynchronous cultures of *P.f* were incubated for 48h with serially diluted fractions combined with lumefantrine at fixed sub inhibitory concentrations. Parasite viability was assessed using the SYBR Green test. Results are expressed as Means ± SD of at least three independent experiments each in duplicate. The IC_50_ values obtained were compared with the IC_50_ values of the fractions alone (*∗*: significant difference, *p < 0.05*). The reversal ratio and combination indexes were calculated. RR: Reversal Ratio; CE: *Lepidobotrys staudtii* crude extract; HXN: hexane fraction; DCM: dichloromethane fraction; EA: ethyl acetate fraction; BOH: butanol fraction; AQ: aqueous fraction; PPX: precipitate; CI: Combination Index (CI < 1: synergism; CI = 1: additivity; CI > 1: antagonism).

We noted that in *Pf*3D7, the combination of EA and BOH fractions with CQ or DHA showed positive interactions with reversal ratios of about 1.35-fold and 1.26-fold, respectively, while lumefantrine had a positive interaction by enhancing the activity of all fractions with reversal ratios ranging from 1.33 to 4.41 (Table 3, Table 4, Table 5).

The best potentiating activity was observed against *Pf*INDO, where following chloroquine addition, all the fractions had positive interactions with reversal ratios varying from 4.38 18.43. Subsequently, EA or BOH fractions combined with DHA showed a trend similar to that of the *Pf*3D7 strain, with a reversal ratio of ∼1.35. There was a positive enhancement between HXN, DCM, or BOH fractions and LUM, showing reversal ratios varying from ∼ 1-to 2-fold. Interestingly, all fractions combined with a fixed concentration of CQ, which was four times lower than the IC_50_ (50 ng/mL) displayed remarkable potency in the IC_50_ values at sub-nanomolar concentrations (Table 3, Table 4, Table 5).

### 3.6 Protective effect of plant fractions on ROS generation in *P. falciparum* INDO culture

**Fig. 5:**
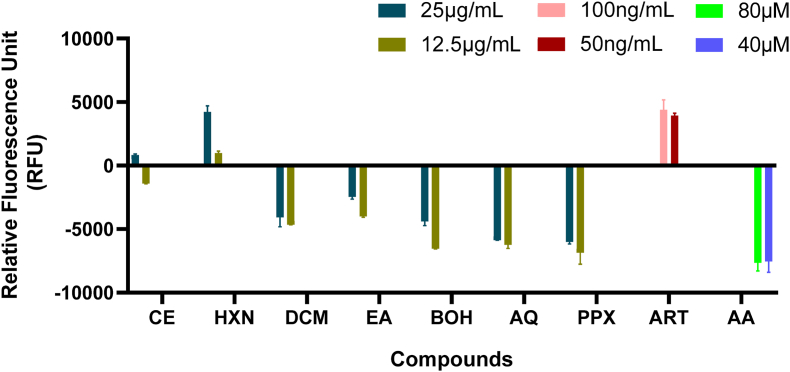
Reactive oxygen species generation in *Plasmodium falciparum* INDO. Parasitized erythrocytes were labelled with DCF-DA in the dark for 30min. After washing, they were incubated for 6 h with crude extract, fractions, ART or ascorbic acid. The level of ROS in the infected erythrocytes was detected by DCF measurements. The results are expressed as the mean ± SD of two different experiments each in triplicate.

The antioxidant properties of the crude extract and fractions were demonstrated by measuring reactive oxygen species at the trophozoite parasite stage. As showed in Fig. 5, culture treatment with DCM, AE, BOH, AQ, PPX (25-12.5 μg/mL) and ascorbic acid (80-40 μM) resulted in a concentration-dependent reduction in fluorescence intensity. However, treatment with the HXN fraction (25-12.5 μg/mL) and ART (100-50 ng/mL) increased fluorescence intensities in a concentration-dependent manner. This suggests that DCM, AE, BOH, AQ, and PPX fractions reduced the amount of ROS produced in *Plasmodium falciparum-*infected cultures.

### 3.7 Phenotypic analysis by the ethyl acetate fraction against *P. falciparum* INDO

Time-kinetic and stage-specific studies have shown that the EA fraction preferentially acts on the ring and schizont stages during intra-erythrocytic development of *P. falciparum*. To support these results, mid-ring (∼10 hpi), early trophozoites (∼20 hpi), or mid-schizonts (∼40 hpi) of *P. falciparum* were directly exposed to the EA fraction at final concentrations of 10 μg/mL (∼2 × IC_50_) or 20 μg/mL (∼4 × IC_50_). The effect of the treatment on the ring and trophozoite stages was monitored every 12h, while the schizont stage was monitored every 4 h for 24 h. The negative control consisted of culture and complete medium.

**Fig. 6:**
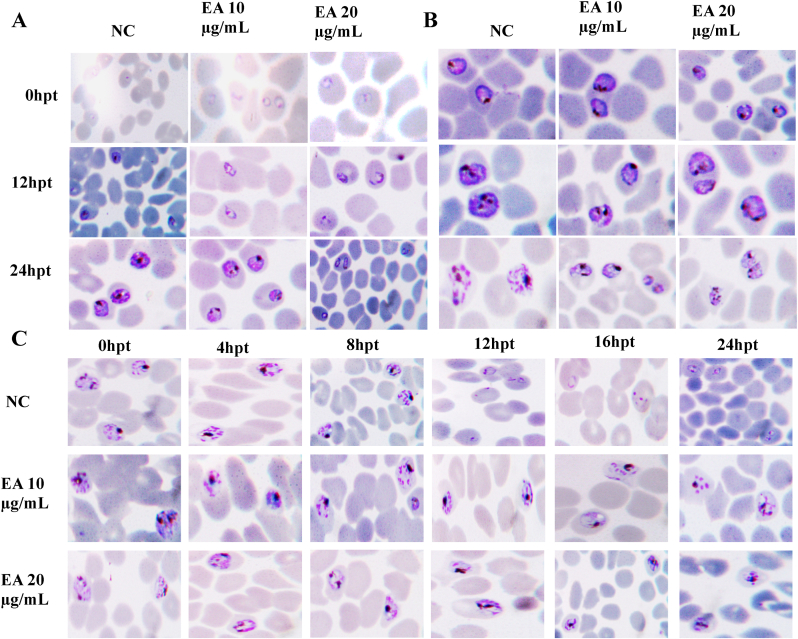
Morphology of *P. falciparum* intra-erythrocytic treated stages. Synchronized *Pf*INDO cultures were treated with ethyl acetate fraction (10 and 20 μg/mL) at rings (A), trophozoites (B) or schizonts (C) stages respectively, and development was monitored for 24 h. Giemsa-stained blood smears were used to observe the morphology of the parasites. EA: ethyl acetate fraction; NC: negative control; hpt: hour post treatment.

**Fig. 7:**
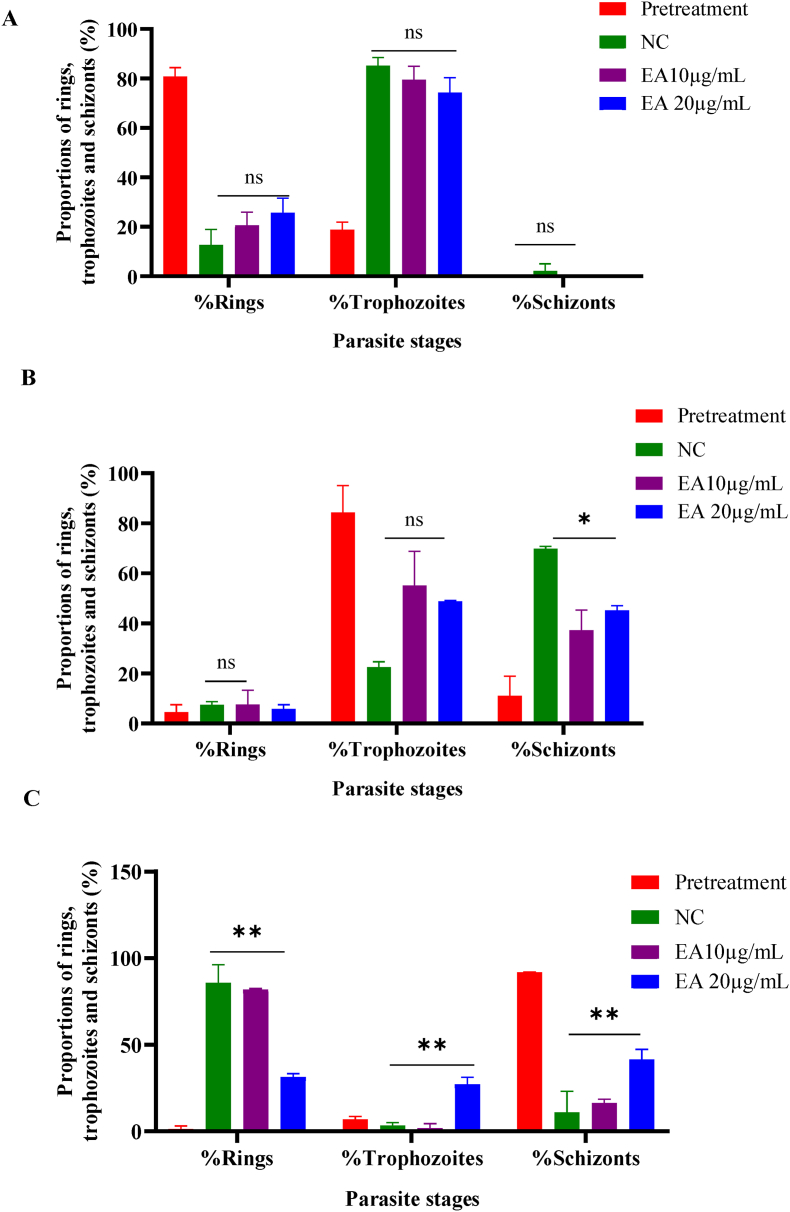
Distribution of *P. falciparum* life cycle stages. *Pf*INDO mid-ring (A), early trophozoites (B) or mid-schizont (C) stage parasites (Pre-treatment) were treated with ethyl acetate and incubated for 24 h. Proportions of each parasite stage were determined and compared with those of the negative control. Bars represents means of 2 independent experiments. EA: ethyl acetate fraction; NC: negative control.

The results showed that the ethyl acetate fraction delayed parasite growth. In the treated cultures, parasites were in the mature ring stage (∼12 hpi) after 12 h of exposure to the middle ring (10 μg/mL), whereas in the negative control, parasites were in the mid-trophozoite stage (∼30 hpi). After 24 h of exposure, in comparison with the negative control, which presented mature trophozoites (∼34 hpi), parasites treated with 10 μg/mL EA were still in the mid-trophozoite stage (∼30 hpi). At 20 μg/mL, after 24 h of treatment, the parasites were still in the mature ring stage (∼16 hpi) (Fig. 6, Fig. 7A). Similarly, young trophozoite parasites treatment (∼20 hpi) at 10 or 20 μg/mL caused a delay in trophozoite maturation. In fact, after 12 h of exposure, compared with the negative control, where parasites were in the early schizont stage (∼38 hpi), the treated parasites were still at the trophozoite stage. In addition to growth inhibition, 24 h exposure caused parasite degeneration in a dose-dependent manner, which was more pronounced in the 20 μg/mL treated culture than in the 10 μg/mL treated culture (Fig. 6, Fig. 7B).

Schizont-stage treatment (∼40 hpi) at 10 and 20 μg/mL resulted in cessation of parasite growth after 4h of exposure. In the negative control, after 12 h of exposure, parasites were already at the ring stage (a sign that the mature schizonts were able to release merozoites capable of infecting new cells), whereas in the treated cultures, they remained at the schizont stage, showing a deficit in merozoite maturation and liberation. In addition, after 8h of exposure, the fraction began to exert a degenerative effect on parasites. After 24 h of exposure, unlike the negative control and culture treated at 10 μg/mL where the parasites were mainly at the ring stage, parasites were still at the schizont stage in culture treated at 20 μg/mL (Fig. 6, Fig. 7C).

### 3.8 Total phenolic and flavonoid contents of *L. staudtii* crude extract and fractions

Phytochemical screening of the *L. staudtii* crude extract and fractions revealed the presence of the phytoconstituents phenols, flavonoids, proteins, tannins, alkaloids, carbohydrates, glycosides, and saponins (Table S2). Polyphenols were present in all fractions, with contents ranging from 82.92 to 971.48 mgGAE/g extract, while flavonoid contents ranged from 6.84 to 60.96 mgQE/g extract. The EA fraction exhibited the highest total polyphenol and flavonoid contents followed by the butanol fraction and the crude extract with respective values of 971.48, 727.77 and 552.96 mgGAE/g extract for polyphenols and 60.96, 33.16 and 33.84 mgQE/g extract for flavonoids. Flavonoids were not present in the PPX fraction (Table 6).

**Table 6:** Total phenol and flavonoid content of *L. staudtii* crude extract and fractions.

Fractions	TPC (mg GAE/g of extract)	TFC (mg QE/g of extract)
**CE**	552.96 ± 1.69	33.84 ± 0.019
**HXN**	82.92 ± 2.31	14.26 ± 0.03
**DCM**	84.44 ± 2.9	8.25 ± 0.56
**EA**	971.48 ± 1.69	60.96 ± 0.025
**BOH**	727.77 ± 0.69	33.16 ± 0.72
**AQ**	325.18 ± 4.2	6.84 ± 0.06
**PPX**	275.18 ± 3.39	–

The phenols and flavonoid contents were determined by Folin-Ciocalteu and aluminium chloride tests, respectively. Results are expressed as mean ± standard deviation of 2 independent experiments each in triplicate. CE: *Lepidobotrys staudtii* crude extract; HXN: hexane fraction; DCM: dichloromethane fraction; EA: ethyl acetate fraction; BOH: butanol fraction; AQ: aqueous fraction; PPX: precipitate; TPC: Total phenol contents; TFC: Total flavonoid contents. -: absent.

### 3.9 Reverse-phase high performance liquid chromatography analysis

**Fig. 8:**
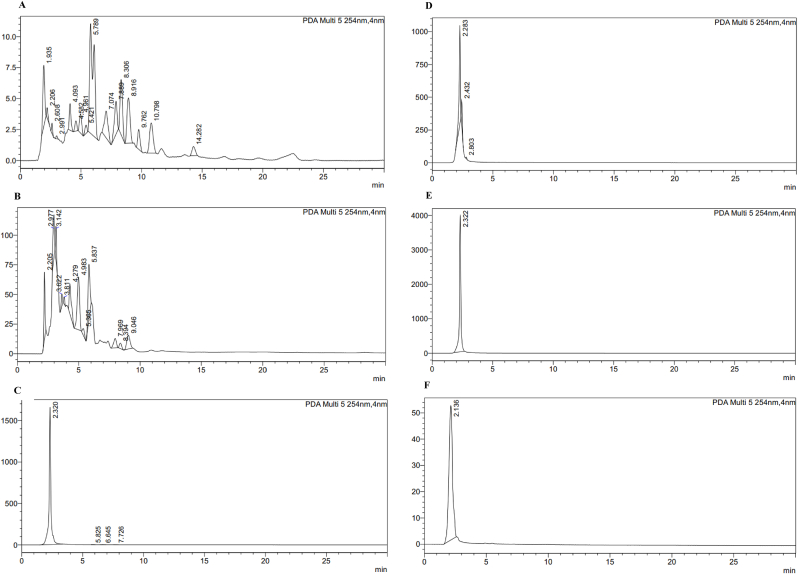
RP-HPLC chromatograms of HXN fraction (A), DCM fraction (B), EA fraction (C), BOH fraction (D), AQ fraction (E) and PPX fraction (F). signal was recorded at 254 nm.

RP-HPLC chromatograms identified a large number of peaks in the fractions, with retention times ranging from 1.93 to 14.28 min (Fig. 8).

The RP-HPLC profile of the HXN fraction revealed 16 peaks with retention times ranging from 1.93 to 14.28 min. The peak with the largest area (33.44 %) had a retention time of 5.78min (Fig. 8A). The chromatogram of the DCM fraction showed 12 peaks with retention times ranging from 2.2 to 9.04min. The peak with the largest area (22.19 %) had a retention time of 4.98 min (Fig. 8B). For the EA fraction, the chromatogram revealed 3 peaks with retention times between 2.28 and 2.8 min, the largest area (83.21 %) being that of the peak with a retention time of 2.28 min (Fig. 8C). For the AQ fraction, we identified four peaks with retention times between 2.32 and 7.72 min. The peak with the largest area (99.87 %) had a retention time of 2.32 min (Fig. 8E). Finally, in each of the chromatograms of the BOH and PPX fractions we observed the presence of a peak with retention times of 2.32 and 2.13 min respectively for areas of 100 % each (Fig. 8D and F).

## 4 Discussion

Despite enormous progress made in the fight against malaria, the disease remains a major public health problem. Resistance developed by parasites against antimalarial drugs is of increasing interest in the search for new active candidates (Amoah et al., 2015). For decades, studies on the search for new antimalarial therapies have made use of numerous Cameroonian medicinal plants, belonging to various families, used by indigenous populations to treat malaria (Kamdoum et al., 2022; Mfopa et al., 2017; Tepa et al., 2022; Kemgne et al., 2012; Keumoe et al., 2021; Dongmo et al., 2023; Jiatsa Mbouna et al., 2022; Bickii et al., 2007). Plants contain secondary metabolites that represent a new source of therapeutic agents that can be used against bacteria, viruses, and parasites (Jiatsa Mbouna et al., 2022). *Lepidobotrys staudtii* belongs to the *Lepidobotryaceae* family, whose bark is used to treat malaria fevers and burns (Tane et al., 1996). However, no scientific study has been conducted to demonstrate its antimalarial properties.

This study determined the antiplasmodial properties of *Lepidobotrys staudtii* stem bark against blood-stage parasites. The time-dependent antiplasmodial activities of the fractions were analyzed using a time-inhibition growth assay. In addition, the stage-specific activity of the fractions, the effects of standard antimalarials on fraction activity, reactive oxygen species generation, and the phenotypic effects of the most potent fraction were assessed. Finally, high-performance thin layer chromatography was used to identify the diverse potential secondary metabolites in these fractions.

Fractionation of the crude extract using liquid-liquid partition yielded polar and non-polar fractions. The crude extract mainly contained polar components (ethyl acetate and butanol fractions with yields of 10.53 % and 34.1 %, respectively), although apolar constituents were also present. The crude extract was prepared by maceration in an ethanol/water mixture (70/30), which is commonly used to extract polar and nonpolar plant compounds (Nureye et al., 2021). Antiplasmodial activities were evaluated in one sensitive (3D7) and two resistant (INDO and Dd2) strains of *P. falciparum* using the SYBR Green I assay.

In this study, the anti-plasmodial potential of the crude extract and fractions was classified based on their IC_50_ values, which states that plant extracts or fractions can be considered highly active (IC_50_ < 5 μg/mL), promising active (5≤ IC_50_ < 15 μg/mL), moderately active (15≤ IC_50_ < 50), and inactive (IC_50_ ≥ 50 μg/mL) (Jonville et al., 2008). Against *Pf*3D7, HXN (IC_50_ = 3.73 μg/mL; SI > 100), DCM (IC_50_ = 4.3 μg/mL; SI = 73.09) and EA (IC_50_ = 3.9 μg/mL; SI > 128.2) fractions showed a high activity while crude extract (IC_50_ = 5 μg/mL; SI > 100) BOH (IC_50_ = 5 μg/mL; SI > 100) AQ (IC_50_ = 7.13 μg/mL; SI > 100) and PPX (IC_50_ = 6.63 μg/mL; SI = 30.99) exhibited promising activities. Against *Pf*INDO, only the ethyl acetate fraction showed a high activity (IC_50_ = 4.8 μg/mL) whereas crude extract ((IC_50_ = 7.3 μg/mL), HXN (IC_50_ = 7.25 μg/mL), DCM (IC_50_ = 5.93 μg/mL), BOH (IC_50_ = 6.6 μg/mL), AQ (IC_50_ = 7.5 μg/mL) and PPX (IC_50_ = 7.85 μg/mL) exhibited promising activities. Screening against *Pf*Dd2 revealed that apart from HNX (IC_50_ = 5.51 μg/mL) and DCM (IC_50_ = 7.33 μg/mL) fractions, which showed promising activities, the crude extract (IC_50_ = 2.02 μg/mL), EA (IC_50_ = 1.62 μg/mL), BOH (IC_50_ = 1.7 μg/mL), AQ (IC_50_ = 3.78 μg/mL) and PPX (IC_50_ = 3.37 μg/mL) fractions showed high activities.

The long-term efficacy of antimalarial drugs depends on their ability to withstand parasite resistance mechanisms. Cross-resistance was assessed by exposing the fractions to the resistant strain *Pf*INDO (resistant to chloroquine) and the multi-resistant strain *Pf*Dd2. Resistance indices comparing the activities of sensitive and resistant strains were all close to 1 and less than 2, respectively, proving that the fractions were active against both strains (Keumoe et al., 2021). These fractions showed better antiplasmodial activity against *Pf*Dd2 than *Pf*INDO or *Pf*3D7, suggesting a novel target in *the Pf*Dd2 strain (Jiatsa Mbouna et al., 2022).

The cytotoxic test on Vero cells revealed that the cytotoxic concentration values for the HXN and DCM fractions were 115.6 and 314.3 μg/mL respectively, and greater than 500 μg/mL for crude extract and other fractions, indicating that the extract and fractions were non-cytotoxic. To confirm the safety of the fractions, selectivity indices (SI) were determined, and according to (Chaniad et al., 2022), they were therapeutically safe, as the obtained SI values were all greater than 10. This indicates that *the L. staudtii* fractions specifically affect the parasite developmental growth cycle and are considered relatively safe. Moreover, compounds in this plant are thought to protect cells from the cytopathic effects of HIV (Bokesch et al., 1996). The non-cytotoxic effect was reinforced by the low percentage of erythrocyte lysis when treated with concentrations close to the IC_50_ values of the fractions. Phytochemical analysis of the extracts revealed the presence of numerous secondary metabolites including phenols, flavonoids, tannins, and saponins (Table 3, Table 4, Table 5 and S3). These results are supported by those obtained in previous studies, where terpene and phenolic acid compounds were isolated from the stem bark of *Lepidobotrys staudtii* (Bokesch et al., 1996; Tane et al., 1996). In the present study, the crude extract and fractions showed IC_50_ < 10 μg/mL, RI ≈ 1, and SI > 10. This means that, besides their anti-plasmodial potential, they are non-cytotoxic, with no observed cross-resistance. The most commonly used antimalarial compounds were alkaloids (chloroquine) and terpenes (artemisinin). The observed antiplasmodial activities are thought to be due to the presence of these secondary metabolites.

The development of effective antimalarial drugs with new modes of action requires consideration of a number of parameters, including stage specificity and speed of drug action (Sanz et al., 2012). The speed of action of any antimalarial agent can be categorized as fast acting (inhibitory action within 24 h of exposure), moderately fast acting (inhibitory action within 48 h of exposure), and slow acting (inhibitory action within 72–96 h of exposure (Rajendran and Naveen, 2024; Mariebernard et al., 2022). As shown in Fig. 2 against the sensitive strain, HXN, DCM, EA and BOH fractions were fast-acting whereas with AQ and PPX fractions there was no significant difference between the IC_50_ values obtained with the different incubation times. Against *Pf* INDO, only the EA fraction was fast-acting, whereas the other fractions were slow-acting, with the highest activity observed after 96h of incubation. Interestingly, the EA fraction demonstrated a significant antiplasmodial activity after 12h of exposure to *Pf*3D7 and *Pf*INDO (Fig. 2 and S1). Previous studies revealed that artemisinin, dihydroartemisinin and chloroquine were fast-acting compounds (affecting first generation parasites), while atovaquone, doxycycline and azithromycin were slow-acting (affecting second generation parasites) (Mariebernard et al., 2022; de Souza et al., 2019; Biagini et al., 2012; Nutmakul et al., 2020). Together, these results suggest that no-cross resistance against *Pf*3D7 fractions may have the same molecular targets as the reference compounds, whereas with *Pf*INDO, fractions may have different molecular or cellular targets than those of the standard compounds. Fast-acting antimalarial drugs generally act at the level of the digestive vacuole, where they may act either by inhibiting hemoglobin degradation or by targeting aspartyl or cysteine proteases involved in proteolysis (Mariebernard et al., 2022).

Once in erythrocytes, merozoites develop to a ring stage, then to the more metabolically active and larger trophozoites, and finally to multinucleated schizonts (Mishra et al., 2019). In the present study, stage-specific semi-maximal lethal values were determined to evaluate stage-specific activities of the fractions. Synchronized cultures were exposed to the fractions at the respective ring, trophozoite, or schizont stages for 24 h, and IC_50_ values were determined using the SYBR Green I assay. In both parasite strains, the highest activity of all the fractions was noted at the ring stage (IC_50_ < 5 μg/mL), followed by the schizont stage and then the trophozoite stage. It was also observed only the EA fraction demonstrated the most potent activity against the ring stage was the only fraction with promising (*Pf*3D7: IC_50_ = 8.2 μg/mL; *Pf*INDO: (IC_50_ = 5.77 μg/mL) and high (*Pf*3D7: IC_50_ = 2.47 μg/mL; *Pf*INDO: IC_50_ = 3.03 μg/mL) activities following exposure to the trophozoite and schizont parasites stages respectively (Fig. 3, S2). This shows that this fraction is effective at all stages and may have additional modes of action compared to the other fractions. During intra-erythrocytic development, the paroxysm of metabolism is recorded at the trophozoite and schizont stages, which justifies the fact that they are the main targets during the development of antimalarial drugs. However, because of their sequestration in the vessels due to the cytoadherence phenomenon, these parasitic stages tend to escape clearance (Butler et al., 2023). Anti-malarial compounds targeting the ring stage would prevent the development of these forms and facilitate parasite clearance. To further elucidate the phenotypic effects of EA fraction on *Pf*INDO parasites synchronous parasites at the ring, trophozoite, and schizont stages were exposed to the fractions at two concentrations (representing a 2- or 4-fold increase in IC_50_) and incubated for 24 h. This fraction caused a delay in parasite maturation and inhibited schizont rupture and merozoite release (Fig. 6), which was reflected by the accumulation of ring, trophozoite, and schizont levels at 24 h after treatment (Fig. 7A, B, and 7C). This could be due to a disruption in cysteine or aspartyl protease activity involved in hemoglobin degradation. Studies have shown that hemoglobin degradation starts at the ring stage and peaks at the trophozoite stage (Francis et al., 1997; Xie et al., 2015). Falcipain 2 degrades hemoglobin and cleaves membrane proteins, causing membrane rupture. Cysteine protease inhibitors stop parasite growth, inhibit hemoglobin degradation and membrane proteins, and prevent merozoite release (Lehmann et al., 2014; Melo et al., 2018; Dhawan et al., 2003; Dasaradhi et al., 2005). Therefore, the fractions, especially EA, influenced the cysteine protease activity in *P. falciparum*.

Many heme molecules escape detoxification during the breakdown of hemoglobin. The ferrous iron (Fe^2+^) in these molecules oxidizes to ferric iron (Fe^3+^), releasing electrons that react with oxygen to produce reactive oxygen species, causing oxidative stress (Francis et al., 1997; Gomes et al., 2022), with stress levels proportional to *P. falciparum* infection severity (Vasquez et al., 2021). As trophozoites are highly metabolically active in the intra-erythrocytic cycle (Njomnang Soh et al., 2012), they were used to study the fraction effects on ROS production in infected erythrocytes. The absence of ROS indicates that the fractions inhibited hemoglobin degradation and trapped reactive oxygen species, such as ascorbic acid. The radical-capturing ability of the fraction may stem from their flavonoids, which neutralize ROS or inhibit free radical-generating enzymes (Akbari et al., 2022).

Drug combinations identify partner drugs to reference antimalarial drugs for combating resistance (Kumar et al., 2017) while reducing toxicity at low concentrations(Eid et al., 2012b). To evaluate the combined effects, standard antimalarial drugs with half or quarter IC_50_ values were added to the diluted fractions. Combination studies have shown varied interactions between antimalarial drugs and their fractions. In fact, against *Pf*3D7, determination of the combination indices and isobologram representation following combination with CQ or DHA revealed antagonistic interactions with all fractions, whereas with lumefantrine, only fractions EA, BOH, AQ, and PPX had antagonistic effects (Table 3, Table 4; Fig. 4). This suggests that, within the combination, the action of one compound inhibits the effect of the other. Consequently, the molecular targets of these fractions were identical to those of CQ or DHA. Against *Pf*INDO, application of chloroquine to the fractions revealed synergistic effects that significantly lowered IC_50_ values. This synergy was also evident when lumefantrine was combined with the crude extract and the HXN and DCM fractions. Conversely, antagonistic interactions were observed between lumefantrine and certain fractions, specifically EA, BOH, AQ, and PPX. (Table 4, Table 5; Fig. 4). The same observations were made with isobologram analysis, where synergy was represented by points below the additivity line, while antagonism was represented by points above the additivity line (Fig. 4). The synergy observed here would mean that the molecular targets of the fractions would be different from those of the standard antimalarial drugs, and that the partner molecules would combine their respective powers to boost antiplasmodial activity. Evaluation of the antiplasmodial activity of the fractions showed that they were more active against chloroquine-sensitive (*Pf*3D7) than against the resistant strain (*Pf*INDO). The ability of chloroquine to interact with the fractions revealed antagonistic effects with the chloroquine-sensitive strain, whereas synergistic effects were observed against the chloroquine-resistant strain. Based on these results, it can be suggested that the resistant strain was more sensitive to the combination of fractions and chloroquine. This study revealed that chloroquine resistance could be overcome by combining chloroquine with certain medicinal plants. These results are in line with those that revealed that *Hernandia voyronii*, *Careya arborea*, and *Cannabis sativa* bark could reverse the resistance observed against *Plasmodium falciparum-resistant* strains FCM29C1 and MRC-Pf-43 and had a synergistic effect with chloroquine when administered at fixed subinhibitory concentrations (Ratsimamanga‐Urverg et al., 1994; Chenniappan and Kadarkarai, 2010).

Studies have shown that 4-aminoquinoline and amino alcohols prevent hemozoin formation by intercalating between heme molecules in the digestive vacuole, where their protonated forms are trapped. The resistance mechanism is linked to reduced compound concentration in parasites(Wicht et al., 2020). The combination of fractions and chloroquine would have contributed to resistance inhibition.

RP-HPLC analysis was performed to better characterize the plant fractions. In our study, the majority of the peaks were recorded in the HXN (16) and DCM (12) fractions (Fig. 8, S3, Table S4). The majority of *L. st*. compounds are therefore found in these two fractions. The peak with the largest area (5325615 mAU/min) was recorded for the EA fraction. Therefore, the majority of compounds in the plant would be compounds of medium polarity. However, these compounds were not identified because of the lack of a standard containing compounds with retention times similar to those of the fraction. In an earlier study, it was discovered that the leaves and stem bark extracts of *L. staudtii* were rich in three novel dihydroxy-3-friedelanone triterpenes, which are likely responsible for the plant's antiplasmodial properties. Thorough investigation is essential to discover and detail these additional compounds. These compounds are likely to be part of the phenolic group, as phytochemical screening has shown that these fractions are predominantly polyphenols. (Table 4, Table 5).

## 5 Conclusion

This study demonstrates that the stem bark of *Lepidobotris staudtii* and its fractions possess antiplasmodial properties that are effective against the blood stage of malaria. Among these, the ethyl acetate fraction showed the highest antiplasmodial activity. The fractions of *L. staudtii* displayed rapid activity against both sensitive and resistant strains of the parasite, significantly impacting the ring stage. Combining these fractions with CQ significantly decreased the viability of chloroquine-resistant *Pf*INDO parasites, indicating that these fractions might help overcome CQ resistance. Considering these findings, *L. staudtii* has emerged as a promising antimalarial resource, particularly in regions where malaria is endemic. Nonetheless, further research is necessary to explore its inhibitory effects on liver-stage development and its potential against gametocytes.

## CRediT authorship contribution statement

**Jeannette Nina Magoudjou Pekam:** Visualization, Validation, Software, Resources, Methodology, Investigation, Conceptualization. **Noella Molisa Efange:** Writing – review & editing, Methodology. **Lakshminarayana Mishro:** Software, Methodology. **Rodrigue Keumoe:** Writing – review & editing, Methodology. **Bruno Lenta Ndjakou:** Methodology. **Lawrence Ayong:** Validation, Supervision, Funding acquisition, Conceptualization. **Frédéric Nico Njayou:** Writing – review & editing, Validation, Supervision, Conceptualization. **Paul Fewou Moundipa:** Writing – review & editing, Supervision, Formal analysis, Conceptualization. **Vinoth Rajendran:** Writing – review & editing, Writing – original draft, Validation, Supervision, Project administration, Methodology, Funding acquisition, Conceptualization.

## Fundings

J.N.M.P extends gratitude to the DBT-UNESCO-TWAS fellowship (FR number: 3240327702) at Pondicherry University under the guidance of V.R. L.A which is supported by the Institute Pasteur International Network. The V.R. laboratory is supported by research grant from the Department of Science & Technology (DST), INSPIRE-Faculty Project (DST/INSPIRE/04/2018/003541), Ministry of Science and Technology, Government of India.

## Declaration of competing interest

The author is an Editorial Board Member/Editor-in-Chief/Associate Editor/Guest Editor for *International Journal for Parasitology: Drugs and Drug Resistance* and was not involved in the editorial review or the decision to publish this article.

The authors declare the following financial interests/personal relationships which may be considered as potential competing interests: The authors declare that they have no conflicts of interest.

## Data Availability

The data will be made available upon request.
