# Supplementary material for: In vitro antiplasmodial activities of extract and fractions from Lepidobotrys staudtii against sensitive and resistant blood-stage parasites of Plasmodium falciparum
Source: Int J Parasitol Drugs Drug Resist. 2025 Aug 25;29:100610. doi: 10.1016/j.ijpddr.2025.100610 (PMC12451174; doi:10.1016/j.ijpddr.2025.100610)
Supplement: Multimedia component 1 [file mmc1.docx]

**Supplementary data:**

**Table S1: Lepidobotrys staudtii stem bark and leaves isolated compounds**

| Plant parts | Extraction solvent | Isolated compounds | References |
| --- | --- | --- | --- |
| Stem bark | Methylene chloride/methanol (1:1); Methanol | Methyl 3,4,5-tri-O-galloylquinate; 1,3,4,5-tetra-O-galloylquininc acid; 3,4,5-tri-O- galloylquininc acid | [16] |
| Leaves | Methylene chloride | 2α,29-dihydroxy-3-friedelanone; 2β,21α- dihydroxy-3-friedelanone; 6β,21α- dihydroxy-3-friedelanone, 21α-hydroxy-3-friedelanone; 30- hydroxy-3-friedelanone; 29- hydroxy-3-friedelanone; 28- hydroxy-3-friedelanone | [18] |
| Stem bark | Methylene chloride | 2α,29-dihydroxy-3-friedelanone acetyl; 2β,21α- dihydroxy-3-friedelanone acetyl; 2α,29-dihydroxy-3-friedelanone diacetate; 21α-hydroxy-3-friedelanone acetyl; 30- hydroxy-3-friedelanone acetyl; 2α,29-dihydroxy-3-friedelanone diacetate; 2β,21α- dihydroxy-3-friedelanone diacetate; 21α-hydroxy-3-friedelanone acetate; 30- hydroxy-3-friedelanone acetate. |  |

**Phytochemical screening**

**Table S2:** Standard methods for the phytochemical screening.

| **Phytochemical Constituents** | **Phytochemical Test** | **Method** | **Observation** |
| --- | --- | --- | --- |
| Carbohydrates | Molisch’s Test | To the aqueous extract was added 2 drops of Molisch’s reagent and gently pour along the sides of the test tube Conc. H_2_SO_4_ and keep the tubes erect for some times. | Result of violet or purple colour ring indicates test is positive. |
|  | Fehling’s Test | Take 2ml Two millilitres of Fehling’s solution A and B were well mixedin a test tube, and boil. One milliliter of the sample solution was added drop by drop and boil simultaneously. | Formation of Red precipitation indicates test is positive |
| Protein | Xanthoproteic test | In a test tube containing three millilitres of concentrated HNO_3_ was slowly added aqueous extract along the test tube. | Dark yellow or Orange colours indicates that test is positive. |
|  | Ninhydrin test | Extract was mixed with 2mL of 0.2% solution of Ninhydrin, | Appearance of Violet colour indicates the presence of amino acid and proteins |
| Alkaloids | Wagner’s Test | A small amount of extract was dissolve into its solvent and Wagner’s reagent was added. | Formation of Brown or Reddish Brown precipitates indicates that test is positive. |
|  | Hager’s test | Few drops of hagers reagent were added to the 2mL of the plant extract. | Appearance of bright yellow precipitate formation indicates the existence of the alkaloids |
| Glycosides | Salkowski Test | Extract was dissolved in 3ml of chloroform and shaken with 3ml H_2_SO_4_. | Formation of Reddish brown Colour indicates presence of steroidal ring, the glycone portion of the glycoside. |
| Flavonoids | Alkaline reagent test | Crude extract was mixed with 2mL of 2% solution of NaOH. | Formation of Yellow colour and disappearance after adding acid indicates test is positive. |
|  | Lead aceate test | One millilitre of filtrate extract was mixed to few drops of a 10% Lead acetate solution. | Formation of yellow precipitate confirms the presence of flavonoids. |
|  | Shinoda Test | Crude extract was mixed with few fragments of magnesium ribbon and add concentrated HCl dropwise. | Appearance of pink scarlet colour indicates that test is positive. |
| Phenol | FeCl_3_ Test | In a test tube, three drops of FeCl_3_ (5% v/v) were added to 5 drops of sample solution. | Formation of dark green or bluish colour indicates that test is positive. |
|  | Alkaline test | To 1ml of sample were added 3ml of dilute NaOH, the sample turns yellow. Add dilute HCl, yellow colour disappears. | Formation of Yellow colour and disappearance after adding acid indicates test is positive. |
|  | Lead aceate test | 5mL of plant extract (filtrate) were mixed with 3mL of 10% lead acetate solution. | Appearance of milky white precipitate indicates the presence of Phenolic compounds. |
| Saponins | FrothTest | One millilitre of sample was added to 2ml of distilled water and shake well | Observation of Honeycomb froth indicates that test is positive. |
| Tannins | FeCl_3_ Test | FeCl_3_ (0.1% w/v) was added to 1ml of sample solution taken in a test tube | Result of Blue or Greenish colour indicates that test is positive. |

**Table S3**: Phytochemical composition of *Lepidobotrys staudtii* crude extract and fractions.

| **Tests** | | **Samples** | | | | | | |
| --- | --- | --- | --- | --- | --- | --- | --- | --- |
|  |  | **CE** | **HXN** | **DCM** | **EA** | **BOH** | **AQ** | **PPX** |
| Carbohydrates | Fehling’s | + | - | - | - | + | + | - |
|  | Molisch’s | ++ | - | + | ++ | ++ | ++ | ++ |
| Phenols | Lead acetate | +++ | +++ | ++ | +++ | +++ | ++ | + |
|  | Fecl_3_ | +++ | ++ | ++ | +++ | +++ | +++ | +++ |
|  | Alkaline test | - | - | - | + | ++ | +++ | +++ |
| Flavonoids | Lead acetate | +++ | +++ | ++ | +++ | +++ | + | - |
|  | Shinoda | ++ | - | - | ++ | ++ | + | - |
|  | Alkaline test | - | - | - | - | + | + | + |
| Alkaloids | Hager’s | - | + | - | - | - | - | - |
|  | Wagner’s | - | + | - | - | - | - | - |
| Glycosides | Salkowski’s | +++ | ++ | ++ | ++ | ++ | ++ | + |
| Proteins | Ninhydrin | - | - | - | - | - | - | - |
|  | Xanthoprotic | - | + | - | ++ | ++ | ++ | + |
| Tannin | Braymer’s | +++ | ++ | ++ | +++ | +++ | +++ | +++ |
| Saponin | Foam test | +++ | + | +++ | +++ | +++ | + | + |

Secondary metabolites were detected in samples using various tests. CE: *Lepidobotrys staudtii* crude extract; HXN: hexane fraction; DCM: dichloromethane fraction; EA: ethyl acetate fraction; BOH: butanol fraction; AQ: aqueous fraction; PPX: precipitate. +: presence of compound; -: absence of compound.

**Table S4: HPTLC fingerprint data of *Lepidobotrys staudtii* crude extract and fractions**

| Samples | **Developed plate (254 nm)** | **Developed plate (366 nm)** | **Derivatized plate at White light** |
| --- | --- | --- | --- |
| CE | (**9**) 0.08, 0.17, 0.22, 0.32, 0.36, 0.39, 0.42, 0.47, 0.76 | (**14**) 0.05, 0.08, 0.10, 0.13, 0.16, 0.23, 0.27, 0.33, 0.3, 0.40, 0.43, 0.46, 0.58, 0.68 | (**15**) 0.08, 0.14, 0.17, 0.20, 0.22, 0.31, 0.35, 0.39, 0.42, 0.45, 0.47, 0.61, 0.65, 0.69, 0.78 |
| HXN | (**9**) 0.38, 0.40, 0.43, 0.46, 0.48, 0.61, 0.67, 0.72, 0.77 | (**7**) 0.05, 0.09, 0.11, 0.13, 0.46, 0.50, 0.69 | (**16**) 0.09, 0.19, 0.22, 0.28, 0.32, 0.37, 0.41, 0.46, 0.50, 0.54, 0.61, 0.65, 0.69, 0.75, 0.83, 0.90 |
| DCM | (**13**) 0.11, 0.14, 0.17, 0.22, 0.27, 0.30, 0.32, 0.35, 0.37, 0.40, 0.42, 0.45, 0.48 | (**10**) 0.05, 0.08, 0.11, 0.20, 0.24, 0.28, 0.31, 0.39, 0.45, 0.49 | (**15**) 0.09, 0.11, 0.14, 0.17, 0.19, 0.22, 0.26, 0.29, 0.35, 0.40, 0.42, 0.45, 0.49, 0.62, 0.67 |
| EA | (**4**) 0.20, 0.23, 0.33, 0.36 | (**7**) 0.08, 0.10, 0.16, 0.20, 0.23, 0.30, 0.36 | (**6**) 0.07, 0.11, 0.14, 0.18, 0.20, 0.49 |
| BOH | (**3**) 0.06, 0.09, 0.23 | (**5**) 0.06, 0.10, 0.14, 0.20, 0.23 | (**3**) 0.04, 0.07, 0.49 |
| AQ | (**2**) 0.10, 0.23 | (**5**) 0.07, 0.11, 0.18, 0.23, 0.79 | (**2**) 0.05, 0.49 |
| PPX | (**2**) 0.11, 0.24 | (**5**) 0.05, 0.09, 0.12, 0.19, 0.23 | (**2**) 0.04, 0.50 |

CE: *Lepidobotrys staudtii* crude extract; HXN: hexane fraction; DCM: dichloromethane fraction; EA: ethyl acetate fraction; BOH: butanol fraction; AQ: aqueous fraction; PPX: precipitate; (): total numbers of spots.

**Figure S1**: Dose-response curves of stage specific activities of fractions on rings (A), trophozoites (B), schizonts (C) stages against *Pf*3D7 and *Pf*INDO

*Pf*3D7

**A**

**G**

**F**

**E**

**C**

**B**

**D**

**Figure S2:** Dose-response curves of time kinetic activities of crude extract (A), hexane (B), dichloromethane (C), ethyl acetate (D), butanol (E), aqueous (F) and precipitate (G) fractions against *Plasmodium falciparum* sensitive and resistant strains.

**HPTLC analysis**

White light 254nm 366nm Derivatization


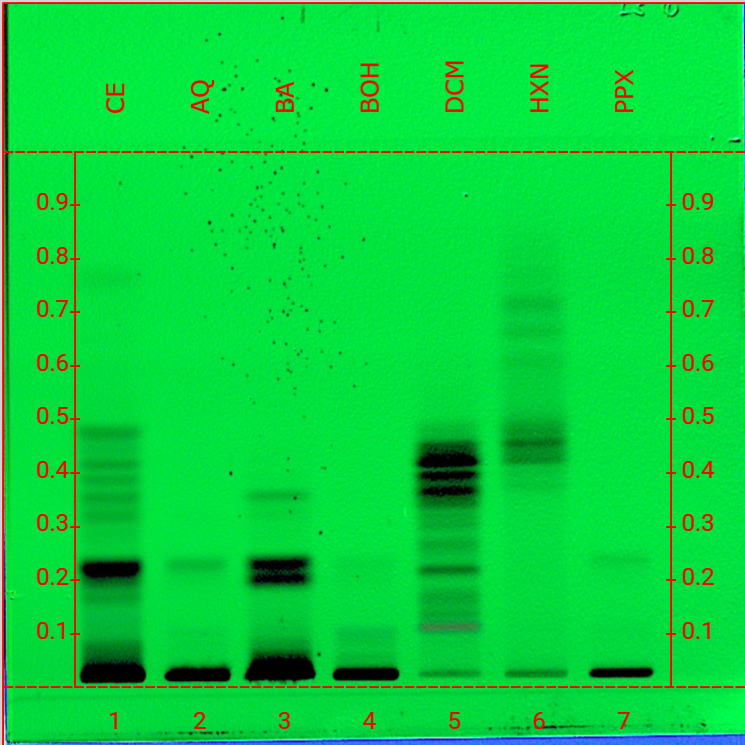


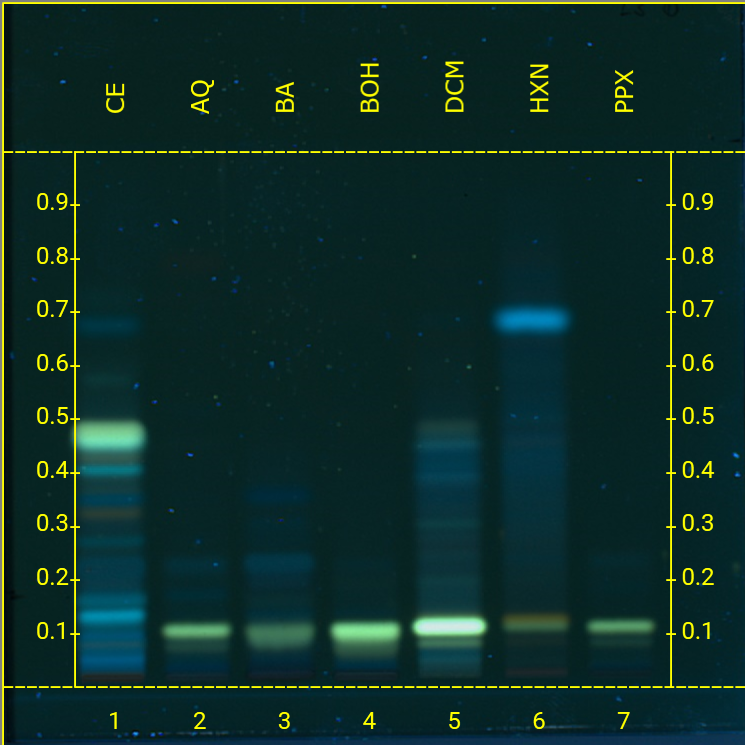


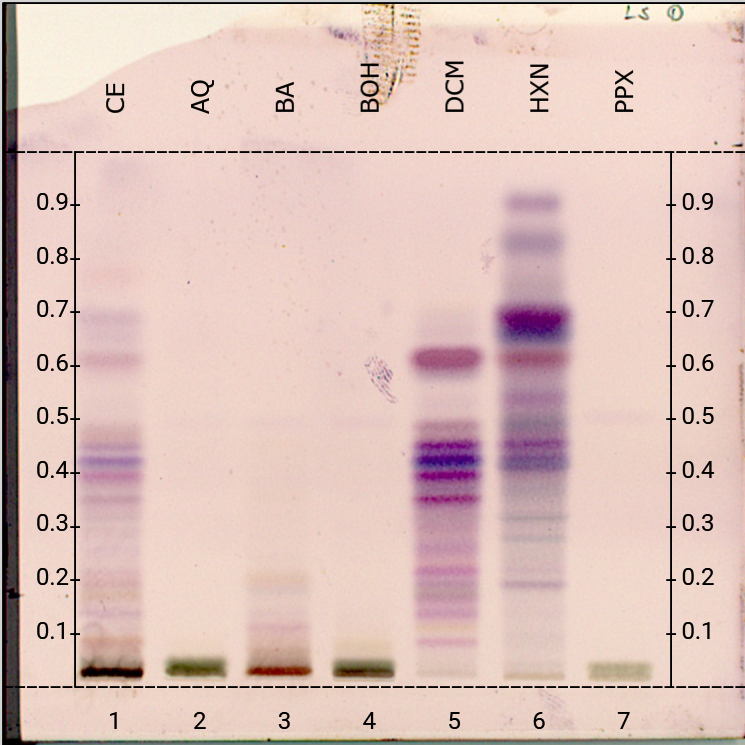


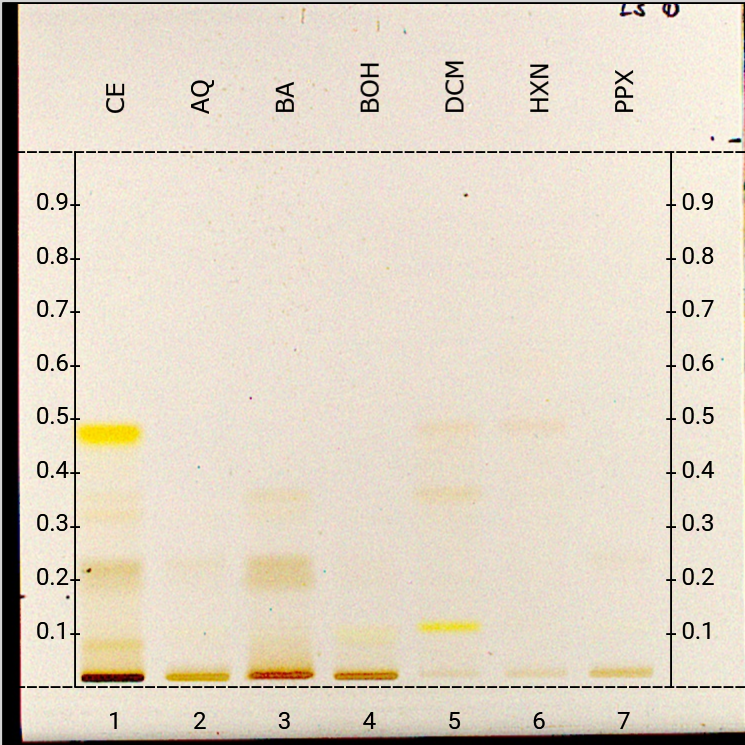


**Figure S4**: HPTLC fingerprint of *Lepidobotrys staudtii staudtii* crude extract and fractions.
